# Cellular N-myristoyltransferases play a crucial picornavirus genus-specific role in viral assembly, virion maturation, and infectivity

**DOI:** 10.1371/journal.ppat.1007203

**Published:** 2018-08-06

**Authors:** Irena Corbic Ramljak, Julia Stanger, Antonio Real-Hohn, Dominik Dreier, Laurin Wimmer, Monika Redlberger-Fritz, Wolfgang Fischl, Karin Klingel, Marko D. Mihovilovic, Dieter Blaas, Heinrich Kowalski

**Affiliations:** 1 Center for Medical Biochemistry, Max F. Perutz Laboratories (MFPL), Medical University of Vienna, Vienna Biocenter (VBC), Vienna, Austria; 2 Institute of Applied Synthetic Chemistry, TU Wien, Vienna, Austria; 3 Center for Virology, Medical University of Vienna, Vienna, Austria; 4 Haplogen GmbH, Vienna, Campus Vienna Biocenter, Vienna, Austria; 5 Cardiopathology, Institute for Pathology and Neuropathology, University Hospital Tübingen, Tübingen, Germany; University of Maryland, UNITED STATES

## Abstract

In nearly all picornaviruses the precursor of the smallest capsid protein VP4 undergoes co-translational N-terminal myristoylation by host cell N-myristoyltransferases (NMTs). Curtailing this modification by mutation of the myristoylation signal in poliovirus has been shown to result in severe assembly defects and very little, if any, progeny virus production. Avoiding possible pleiotropic effects of such mutations, we here used pharmacological abrogation of myristoylation with the NMT inhibitor DDD85646, a pyrazole sulfonamide originally developed against trypanosomal NMT. Infection of HeLa cells with coxsackievirus B3 in the presence of this drug decreased VP0 acylation at least 100-fold, resulting in a defect both early and late in virus morphogenesis, which diminishes the yield of viral progeny by about 90%. Virus particles still produced consisted mainly of provirions containing RNA and uncleaved VP0 and, to a substantially lesser extent, of mature virions with cleaved VP0. This indicates an important role of myristoylation in the viral maturation cleavage. By electron microscopy, these RNA-filled particles were indistinguishable from virus produced under control conditions. Nevertheless, their specific infectivity decreased by about five hundred fold. Since host cell-attachment was not markedly impaired, their defect must lie in the inability to transfer their genomic RNA into the cytosol, likely at the level of endosomal pore formation. Strikingly, neither parechoviruses nor kobuviruses are affected by DDD85646, which appears to correlate with their native capsid containing only unprocessed VP0. Individual knockout of the genes encoding the two human NMT isozymes in haploid HAP1 cells further demonstrated the pivotal role for HsNMT1, with little contribution by HsNMT2, in the virus replication cycle. Our results also indicate that inhibition of NMT can possibly be exploited for controlling the infection by a wide spectrum of picornaviruses.

## Author summary

Picornaviruses are important human and animal pathogens. Protective vaccines are only available against very few representatives. Furthermore, antiviral drugs have not made it to the market because of serious side effects and viral mutational escape. We here show that pharmacological inhibition of cellular myristoyltransferases severely decreased myristoylation of enteroviral structural proteins as exemplified by coxsackievirus B3, a prominent pathogen causing virus-induced acute and chronic heart disease. The drug DDD85646 substantially diminished virus yield and almost abolished the infectivity of the residual progeny virus. It is highly effective against several other picornaviruses, except those two included in our study that naturally do not process VP0. Our work provides new insight into the role of myristoylation in the life cycle of picornaviruses and identifies the responsible cellular enzyme as a promising candidate for antiviral therapy.

## Introduction

The family *Picornaviridae* encompasses 35 genera of small non-enveloped RNA viruses that include numerous animal and human pathogens (www.picornaviridae.com). They are causative agents of diseases with huge impact on health care and economy ranging from the relatively harmless common cold typically caused by rhinoviruses to flaccid paralysis as live-threatening complication from poliovirus infection. Vaccines are available only against few members and currently no antiviral drug has been approved for clinical use [1–3].

The ~30 nm diameter icosahedral picornavirus capsid is—in most instances—built from 60 copies of viral proteins VP1, VP2, VP3, and VP4 (buried inside). It contains a 6.7 to 9.7 kb single-stranded positive-sense RNA genome (ss(+)RNA), whose single open reading frame is translated into a large polyprotein comprising a structural (P1) and two nonstructural domains (P2 and P3). These are separated from each other and further cleaved by virus-encoded proteases into about a dozen smaller proteins required for replication of the viral RNA and capsid assembly. Processing of the P1 precursor by the viral proteases 3C^pro^/3CD^pro^ yields the capsid proteins VP0, VP1 and VP3, which remain associated as a 5S protomer. Five protomers assemble into a 14S (immature) pentamer and twelve pentamers construct the 75S to 80S empty procapsid. Progeny ss(+)RNA produced in replication complexes located on the surface of virus-induced vesicles is either threaded into the procapsid, or more likely, pentamers recruited by the nonstructural protein 2C assemble and condense around the nascent viral RNA. In the latter instance, accumulating procapsids are either regarded as a storage form of pentamers or a dead-end by-product of virion morphogenesis. The resulting 150S provirions undergo autocatalytic cleavage of VP0 into VP2 and VP4 giving rise to infectious virions. Capsid maturation is rapid in enteroviruses (e.g. completed after 1 h in rhinovirus 16 [4]) but quite protracted for hepatoviruses (at neutral pH) [5]. Parecho-, kobu- and saliviruses lack a maturation cleavage and maintain a shell composed of VP1, VP3, and VP0 (reviewed in [6]).

The polyprotein of almost all picornaviruses and derived processing products sharing the same N-terminus (i.e. P1, VP0, VP4) are N-myristoylated [7]. This predominantly co-translational modification involves the transfer of myristate, a 14-carbon saturated fatty acid (C14:0), from myristoyl-CoA to an N-terminal glycine in the context of a myristoylation signal (GXXX(S/T)) following removal of the initiator methionine from the nascent polypeptide chain by methionine aminopeptidase. In apoptotic cells, certain proteins undergo post-translational myristoylation upon exposure of a cryptic glycine by caspase-mediated cleavage [8]. A substantial number of viral and cellular proteins are N-terminally myristoylated, which contributes to their subcellular location and function by modulating protein-membrane and protein-protein interactions [9–11]. In higher eukaryotes, the reaction is catalyzed by the two ubiquitously expressed N-myristoyltransferase isozymes NMT1 and NMT2 (EC 2.3.1.97). Despite overlapping substrate specificity [12], they exert unique roles in a number of biological processes [13, 14].

The atomic structures of poliovirus 1 (PV1) [7], coxsackievirus B3 (CVB3) [15] and several other enteroviruses ([16] and references therein) reveal a spatially very similar organization of the myristic acid chain covalently attached to the N-terminus of VP4, though C14 up to C11 of the fatty acid may be disordered. The myristoyl groups initially cluster together around the icosahedral 5-fold axis and then splay apart to cradle a twisted parallel β-tube composed of the five VP3 N-termini of a pentamer. Each myristoyl chain interacts with its 5-fold related siblings and additionally engages the VP4 and VP3 N-terminal extensions of adjacent 5-fold related protomers. This network of contacts appears to stabilize the mature (VP0 cleaved into VP2 and VP4) pentamer subunits of the virion. Indeed, mutation of Thr 28 in VP4 of PV1, which forms a conserved hydrogen bond with the carbonyl oxygen of a myristoyl moiety from an adjacent five-fold symmetry-related VP4, resulted in unstable virions in concert with assembly anomalies. Notably, the manifestation of the assembly defect was strongly dependent on the replaced amino acid, which complicated interpretation of the results [17].

Only nonviable mutants were obtained, when N-myristoylation was ablated by site-directed mutagenesis of the glycine at the N-terminus of the PV1 polyprotein. However, no consistent picture has emerged on the underlying cause, which ranged from defects in polyprotein processing and RNA replication to impaired viral morphogenesis at different stages [18–24]. Studies on the *in vitro* and *in vivo* formation of myristoylation-deficient assembly intermediates of foot-and-mouth disease virus (an *Aphthovirus*) showed profoundly different outcomes regarding the lipid´s contribution in this process [25–29]. To overcome the technical difficulties associated with these systems [30, 31], a viable PV1 mutant with about 50% reduced myristoylation was generated [32]. Its analysis demonstrated a kinetic preference for modified protomers in the assembly of immature (VP0 containing) pentamers, suggesting that the myristoyl moiety on VP0 might drive the protomer to pentamer transition. Remarkably, unmodified pentamers were excluded from mature virions but not from procapsids. At variance with these results, mutant PV1 with a substitution of proline either at position 2 or 5 in the consensus GXXXS/T myristoylation signal sequence, while featuring a drastically reduced myristoylation, still yielded an appreciable number of progeny virions [23]. They lacked infectivity, which was interpreted as (additional) requirement for VP4 myristoylation in the early steps of cell entry, possibly uncoating, as previously proposed from a structural analysis of PV1 [7]. However, an effect of the proline by itself has not been excluded by these researchers.

In cardioviruses [33, 34], aphthoviruses [35], and even some rhinoviruses of the *Enterovirus* genus [36] the N-terminal part of VP4 including the attached myristate are disordered. At physiological temperatures, capsids of picornaviruses tend to “breath”, allowing partial exit of the normally internal VP4, which includes the N-terminal myristate [37, 38]. This indicates a considerable degree of plasticity and rather weak interactions of this lipid with internal structures [39], casting some doubt on a prominent capsid stabilizing function.

Apart from targeting mostly PV1, a caveat of these virus mutant studies is that replacement of amino acid residues may per se interfere with assembly and thus compound any effect due to the lack of the myristoyl moiety or of certain lipid-protein interactions. The potential attractiveness of this modification as a drug target with a likely high barrier to the emergence of resistance prompted us to revisit its functional relevance for picornaviruses by orthologous non-mutational approaches. Until now, there is only one preliminary study in this direction, which, interestingly, showed a just moderately decreased infectivity of enterovirus 71 following inhibition of cellular myristoylation by 2-hydroxy myristate (2-HMA), in parallel with a diminished VP0 scission [40].

Here, by gene knock-out in haploid HAP1 cells, we demonstrate a greater importance of NMT1 as to the NMT2 isozyme. By employing the potent pan-NMT inhibitor DDD85646 we found that viral assembly is only partially compromised despite a severely reduced VP0 myristoylation, though CVB3 progeny particles were markedly enriched in unstable noninfectious provirions; this indicates an important role for N-terminal myristoylation in efficient VP0 scission for virus maturation. In contrast, parecho- and Aichi virus, which do not process their VP0, are drug-insensitive. The low specific infectivity of CVB3 still made in the presence of DDD85646 is most likely due to defective viral RNA transfer through the endosomal membrane. Finally, our study identifies NMTs as suitable drug target for combating a broad spectrum of picornaviruses.

## Results

### N-myristoyltransferase 1 and 2 isozymes are differentially required for replication of CVB3

Revisiting the importance of myristoylation in the picornaviral life cycle, we used coxsackievirus B3 (CVB3) as a model and investigated the role of the two N-myristoyltransferase isozymes NMT1 and NMT2, which are ubiquitously expressed in mammalian cells. To this end we assessed the impact of the gene knockout of each of the two human NMT isozymes on viral infection in the near-haploid fibroblastoid human cell line HAP1 [41]. HsNMT1 and HsNMT2 (Hs, homo sapiens) are encoded by single multi-exon genes on human chromosome 17 (*NMT1*; Gene ID: 4836) and chromosome 10 (*NMT2*; Gene ID: 9397), respectively. Three enzymatically active HsNMT1 isoforms of different molecular weight have been described. Two of them may result from alternative splicing or initiation at internal start codons as a consequence of leaky ribosomal scanning ([11] and references therein). By contrast, only a single HsNMT2 protein has been found in human tissue [12].

To abolish expression of all reported HsNMT1 isoforms we created a knockout (KO) HAP1 cell line with a CRISPR/Cas9-induced frame shift mutation in exon 3 of *NMT1*. To eliminate HsNMT2 expression, we similarly introduced a frame shift in the *NMT2* exon 1. Repeated attempts to obtain an *NMT1* exon 3 / *NMT2* exon 1 double-KO cell line failed attesting to the essential nature of myristoylation for long-term cell growth and survival. The morphology of the two KO clones was indistinguishable from wt HAP1 cells (S1A Fig), although the *NMT1* KO cells grew somewhat slower mirroring the effect of siRNA-mediated HsNMT1 knockdown in ovarian cancer cells [13]. Western blotting confirmed the expected lack of the individual isozymes in the respective HAP1 mutants (S1B Fig). As previously reported by others, siRNA knockdown of HsNMT1 in SK-OV-3 human ovarian cancer cells resulted in a 60% increase in HsNMT2 protein [13]. In our HAP1 cells, however, we did not see any compensatory up-regulation of the non-targeted NMT isozyme.

**Fig 1:**
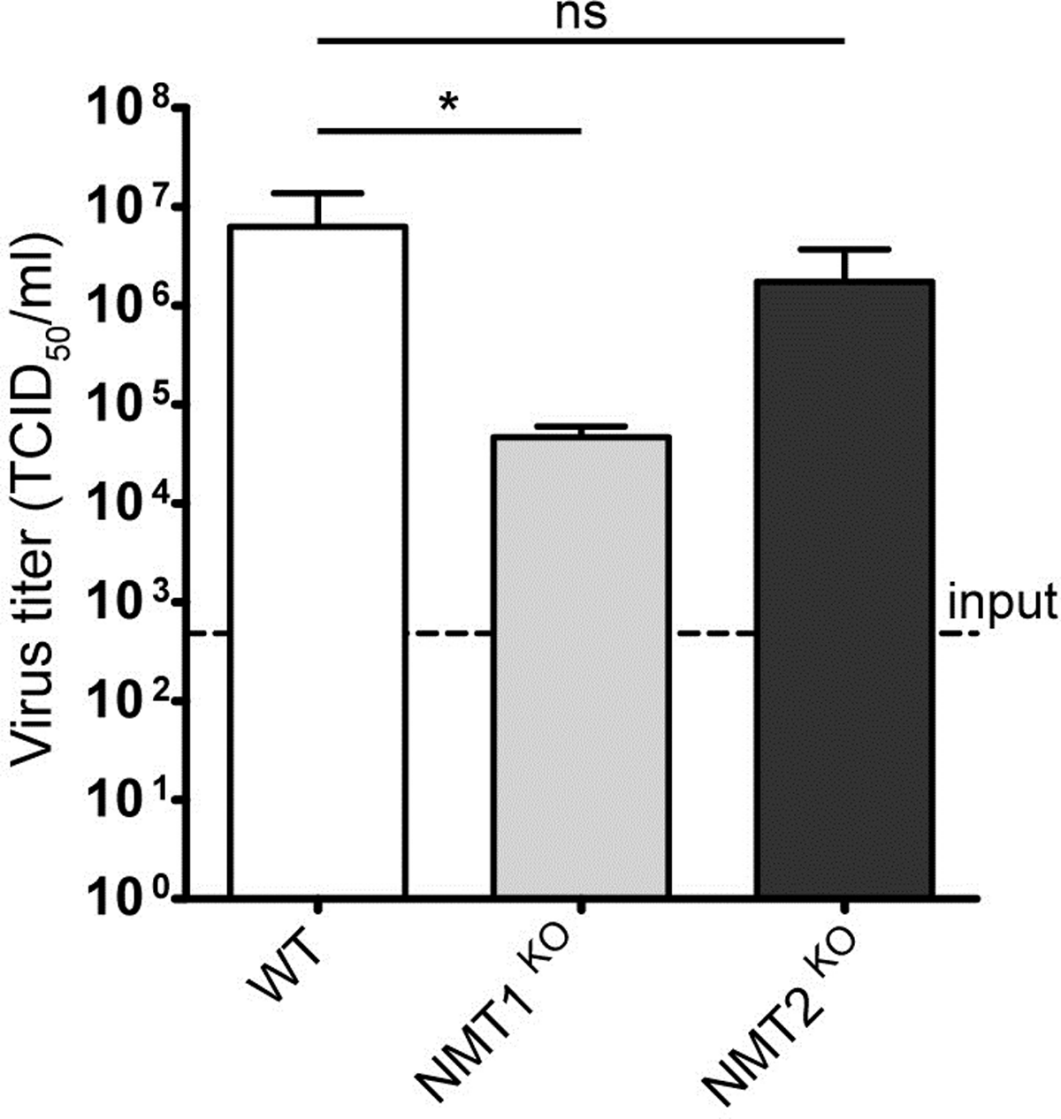
Impact of HsNMT isozymes on infectious CVB3 production. HAP1 cells (wt, NMT1^KO^, NMT2^KO^) were infected with CVB3 at an MOI of 1 and infectious virus titers were measured 7 h p.i. Each bar represents the mean ± SD, n = 3; * p < 0.05 using the two-tailed Student´s t test; ns: not significant.

Lack of HsNMT1 reduced CVB3 production in the mutant HAP1 cells by 2.1 log10 in a single-cycle replication as compared to the parental cells. On the contrary, in HAP1 cells devoid of HsNMT2, the virus titer attained similar values as in wt cells (Fig 1). Reconstitution of HsNMT1 expression in about 80% of HAP1 *NMT1* KO cells (S1C Fig) by transient transfection practically recovered CVB3 production (S1D Fig), indicative of a lack of CRISPR/Cas9 induced off-target effects possibly impacting on virus growth. In summary, the experiments suggested a dominant role of the HsNMT1 isozyme for CVB3 production, at least in HAP1 cells.

### DDD85646, a potent pan-NMT inhibitor, drastically reduces CVB3 titers in various host cells

**Fig 2:**
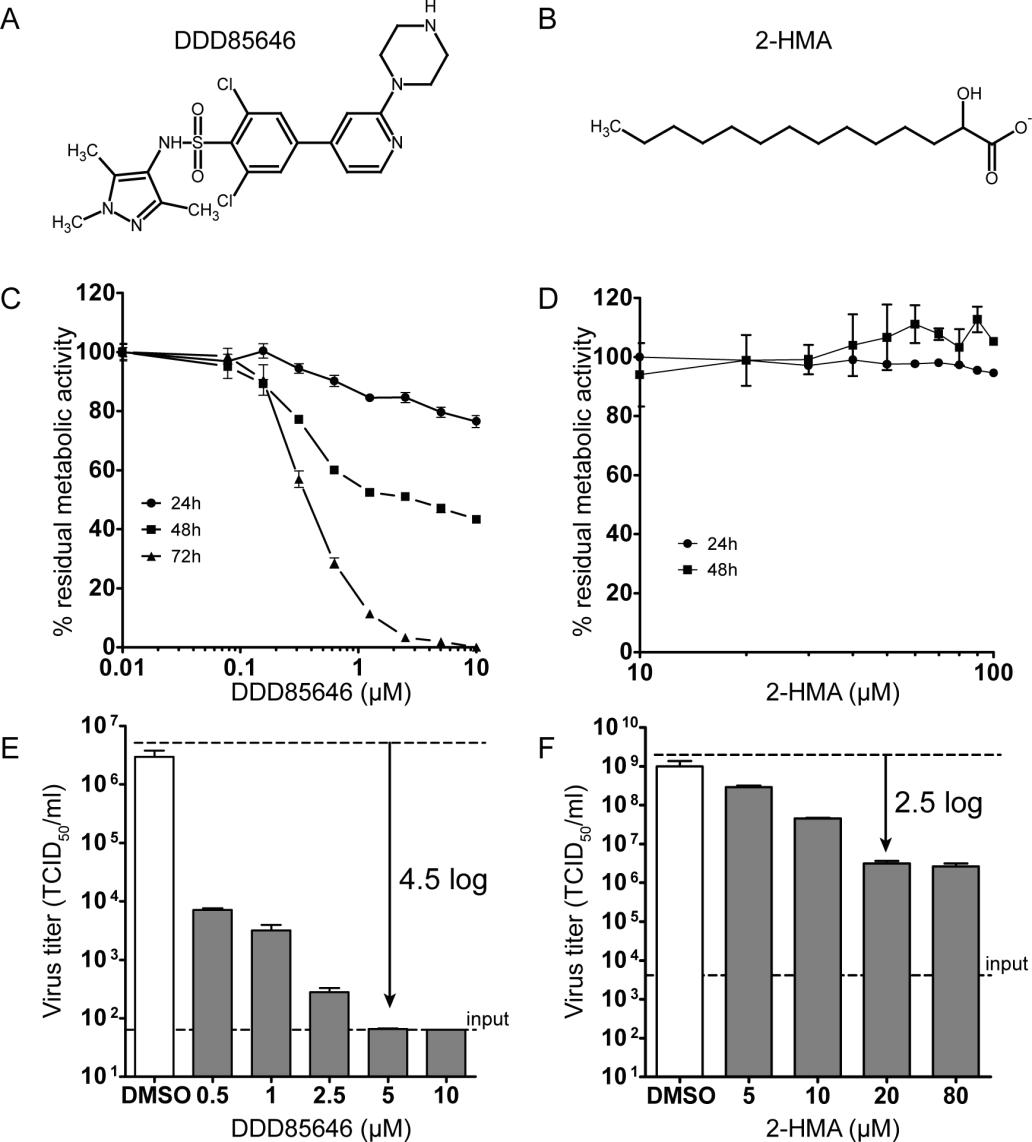
DDD85646 dramatically reduces CVB3 infectious titers. (A) Structural formula of the myristoylation inhibitors DDD85646 and (B) 2-HMA. (C) Effect of DDD85646 and (D) 2-HMA on HeLa cell viability (XTT assay) at the indicated concentrations and times. Each data point represents the mean ± SD, n = 9. (E) HeLa cells were infected with CVB3 at an MOI of 1 and DDD85646 was added 1 h p.i. at the indicated concentrations, with DMSO used as solvent control. Cell lysates prepared 7 h p.i. were used to determine virus yield by end point titration and the data are expressed as the 50% tissue culture infective dose (TCID_50_) per ml. Each bar represents the mean ± SD, n = 3. (F) HeLa cells were infected with CVB3 at an MOI of 1, treated 1 h p.i. with the specified concentrations of 2-HMA and with DMSO as solvent control, lysed and virus titer quantified as before. Each bar represents the mean ± SD, n = 3.

The *NMT1* KO resulted in a substantially decreased production of infectious CVB3, presumably attributable to diminished myristoylation of the viral proteins. However, other effects indirectly perturbing virus replication related to disruption of non-catalytic protein interactions of NMT1 [42–46] and/or ensuing from overall changes of the cellular myristoylome comprising >100 N-myristoylated proteins [47] cannot be excluded. We thus tested DDD85646 for its impact on virus yield. This pyrazole sulfonamide derivative (Fig 2A) inhibits N-myristoylation of trypanosomal proteins [48] and was subsequently validated as a potent HsNMT1/2 inhibitor [47]. The drug competes with peptide substrates with a 50% inhibitory concentration (IC_50_) of ~17 nM (HsNMT1) and ~22 nM (HsNMT2) as determined *in vitro* [49]. For comparison, we used 2-hydroxy myristoic acid (2-HMA; Fig 2B). After cellular uptake, this myristic acid analogue is metabolically converted to the CoA-thioester, which is a potent competitive inhibitor of protein myristoylation (*in vitro* IC_50_ ~50 nM) [50]. 2-HMA was recently found to decrease the titer of enterovirus-71 (EV-71) by about 10-fold at 250–500 μM concentration [40].

In order to rule out that the reduction in virus titer results from a generalized cytotoxic effect of the NMT inhibitors on the cells, we determined HeLa cell viability with the XTT assay over 1 to 3 days of drug treatment (Fig 2C). After 24 h, viability slightly decreased to ~80% at 5 to 10 μM DDD85646. The extrapolated 50% cell cytotoxic concentration (CC_50_) was about 230 μM. Later, cell death increased, thus lowering the CC_50_ to 0.2 μM after 3 days of exposure, essentially as found in a myristoylome profiling study, using this NMT inhibitor [47]. The increase in drug-induced cytotoxicity from day 1 onward likely reflects the turnover of the endogenous pool of myristoylated proteins (e.g. c-Src tyrosine kinase) that become progressively replaced by non-myristoylated counterparts with compromised function [51]. As shown in Fig 2D, 2-HMA was not markedly cytotoxic even after 2 days of incubation, likely due to incomplete inhibition of cellular myristoylation by this compound even at highest achievable doses [52].

HeLa cells were then infected with CVB3 in the absence or presence of increasing concentrations of DDD85646 (up to 5 μM where > 80% of uninfected HeLa cells were still viable) or 2-HMA (up to 100 μM) added 1 h post infection (p.i.) and virus production was measured 7 h p.i. As shown in Fig 2E, DDD85646 reduced the yield of infectious CVB3 progeny in a dose-dependent manner by 4.5 log10 at 5 μM relative to the DMSO-treated solvent control. The 50% effective concentration (EC_50_) was clearly below 0.5 μM, the lowest concentration tested, which still reduced the titer by 2.7 logs. 2-HMA was also effective (EC_50_ = 7.5 μM), but the highest achievable reduction of virus titer was only 2.5 log10 at 20 μM; above this concentration its inhibitory activity quickly plateaued (Fig 2F). The relatively low solubility of 2-HMA paired with a requirement for intracellular conversion to the coenzyme A derivative presumably limits its antiviral effect, as also observed by others [40, 53]. Addition of MA fully relieved the effect of 2-HMA (S2B Fig) but not of DDD85646 in CVB3-infected HeLa cells (S2A Fig) as expected from the NMT´s sequentially ordered bi-bi mechanism [54]. The observed inhibitory properties of these drugs thus agree well with previous *in vitro* results for trypanosomal NMT [48]. At 5 μM, DDD85646 was also active against CVB3 in various other cell lines with two of them frequently employed as models for organs naturally infected by this virus (Caco-2 for intestinal tract enterocytes, A549 for lung epithelial cells; Table 1). In each instance, cell viability was greater than 75% as determined by the XTT assay (S3 Fig). Altogether, this confirms the essential role of HsNMT in CVB3 infectious progeny production.

**Table 1:** Antiviral activity of DDD85646 against various picornaviruses.

Species	Serotype (Strain)	Cell line	Time of sample collection (h p.i)	Titer. DMSO	Titer. DDD85646
**Enterovirus B**	Coxsackievirus B3 (Nancy)	HeLa	7	6.50±0.25	1.82±0.02
	Coxsackievirus B3 (Nancy)	Vero	7	4.64±0.14	2.28±0.24
	Coxsackievirus B3 (Nancy)	A549	7	5.33±0.30	1.77±0.38
	Coxsackievirus B3 (Nancy)	Caco2	7	2.53±0.64	BLQ
	Coxsackievirus B1	HeLa	7	7.87±0.08	5.82±0.25
**Cardiovirus A**	Mengovirus (VMC0)	HeLa	7	8.71±0.03	3.20 ±0.43
**Rhinovirus A**	Rhinovirus A2	HeLa	10	5.14±0.27	2.45±0.22
**Rhinovirus B**	Rhinovirus B14	HeLa	10	9.48±0.04	3.67±0.11
**Rhinovirus C**	Rhinovirus C15	CDHR3-HeLa	8	5.36±0.32	4.09±0.14
**Kobuvirus**	Aichi virus 1	Vero	10	5.66±0.40	5.49±0.29
**Parechovirus**	Human parechovirus 1	Vero	7	4.96±0.36	5.38±0.52

Data are the mean ± SD from at least three independent experiments and are expressed in log10 TCID_50_/ml.

BLQ below limit of quantification.

### Inhibition by DDD85646 correlates with processing of VP0 in picornavirus genera

At 5 μM, DDD85646 was also active against four other enteroviruses tested (Table 1): coxsackievirus B1 (CVB1), representatives of the 3 different rhinovirus species (RV-A2, RV-B14, and RV-C15), as well as against mengovirus, a member of the *Cardiovirus* genus. The drug decreased virus titers by 5.5 log10 (RV-B14) to 2.0 log10 (CVB1) compared to the DMSO control. Strikingly, human parechovirus 1 (HPeV-1, formerly echovirus 22, genus *Parechovirus*) and Aichi virus 1 (AiV-1, genus *Kobuvirus*) were completely insensitive to drug treatment. In both viruses, VP0 remains uncleaved, resulting in their native capsid being built from 60 copies of VP0, VP3 and VP1 [6]. The above results thus suggest that myristoylation is dispensable for the infection cycle of those picornaviruses that lack maturation cleavage of VP0.

### DDD85646 severely reduces incorporation of the myristic acid analogue Alk-12 into VP0

**Fig 3:**
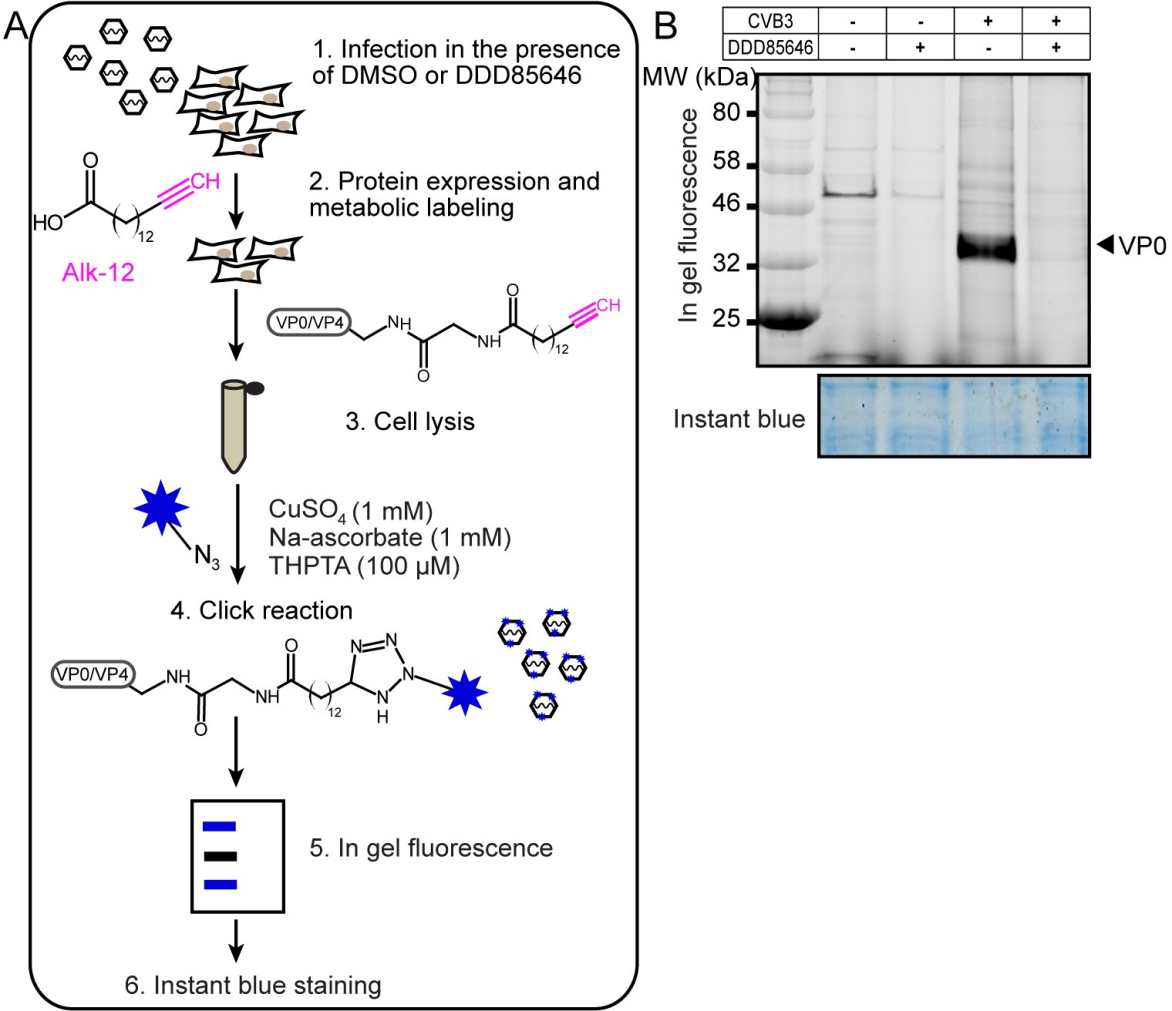
DDD85646 massively reduces metabolic incorporation of the Alk-12 myristate analogue into VP0. (A) Work flow for detection of Alk-12 labeled viral proteins with click reagents. HeLa cells (DDD85646- or DMSO-treated) are infected with CVB3 at MOI of 10. The myristic acid analogue Alk-12 is added 4 h p.i. and incubation continued for 3 h; during this time Alk-12 becomes transferred to viral substrate proteins by metabolic incorporation. Cells are lysed and Alk-12-bearing proteins are ligated to the fluorescent reporter 5-TAMRA-azide by click chemistry, separated by SDS-PAGE and visualized by in-gel fluorescence. (B) DDD85646 at 5 μM blocks incorporation of Alk-12 into CVB3 VP0. The gel subjected to in-gel fluorescence recording was subsequently stained with InstantBlue to visualize total proteins for verifying equal loading.

We next verified whether DDD85646 treatment curtailed myristoylation of the structural proteins VP0/VP4 of CVB3. We utilized metabolic tagging with the Alk-12 myristic acid analogue followed by bioorthogonal ligation of the terminal alkyne moiety to the azido group of the fluorescent reporter 5-TAMRA-azide via Cu^I^-catalyzed azide-alkyne cycloaddition (CuAAC, the so-called “click” reaction) for subsequent in-gel visualization (outlined in Fig 3A). A similar strategy was recently employed in a proteome-wide analysis of myristoylated proteins in herpes simplex virus-infected cells [55]. First, we confirmed that Alk-12 became attached to a variety of HeLa proteins in a NMT-controlled manner by the DDD85646 dose-dependent reduction of their in-gel fluorescence over a 24 h treatment period (S4A Fig). Incorporation of Alk-12 was practically abolished by the drug at 5 μM. Importantly, incorporation of the clickable methionine analogue L-azido-homoalanine demonstrated that endogenous protein synthesis proceeded unperturbed up to the highest drug concentration tested (i.e. 10 μM; S4B Fig). This fits well with the only slightly reduced metabolic activity determined in the XTT assay (see Fig 2C, 24 h exposure to DDD85646).

We then infected HeLa cells with CVB3 at a multiplicity of infection (MOI) of 10 in absence or presence of 5 μM DDD85646 (the concentration, which resulted in practically complete inhibition of infectious virus production) and pulse-labeled with Alk-12 at 4 h p.i. At that time host cell shut-off is already well developed, which should result in preferential incorporation of the alkynyl-myristic acid analogue into viral proteins. Cells were harvested at 6 h p.i. and metabolically tagged proteins visualized by in-gel fluorescence (Fig 3B). In extracts from CVB3-infected cells, a strongly fluorescent band was detected at about 36 kDa that most likely corresponded to VP0 with covalently linked Alk-12. This band almost disappeared when the NMT inhibitor was present during viral infection (compare the two rightmost lanes in Fig 3B), and, accordingly, it was not detected when the cells were sham-infected for control either with or without inhibitor (first two lanes in Fig 3B); in the latter case incorporation of Alk-12 into a cellular protein migrating at about 50 kDa was strongly reduced by DDD85646. By densitometric analysis, the fluorescent VP0 signal decreased ~100-fold at 5 μM of the drug.

The small Alk-12 modified VP4 (7.4 kDa) was not visible due to a substantial fluorescent background obscuring bands in the low molecular weight range (S4C Fig). Regardless of this technical problem, our results clearly demonstrate that DDD85646 effectively inhibits incorporation of Alk-12 into VP0 at the same concentrations that practically eliminate CVB3 infectivity, suggesting a cause-effect relationship. Consequently, we used a 5 μM drug concentration in all subsequent experiments to efficiently suppress myristoylation with minimal impact on cell viability over the testing period.

### HsNMTs are essential for efficient initiation of a second infection cycle by CVB3

**Fig 4:**
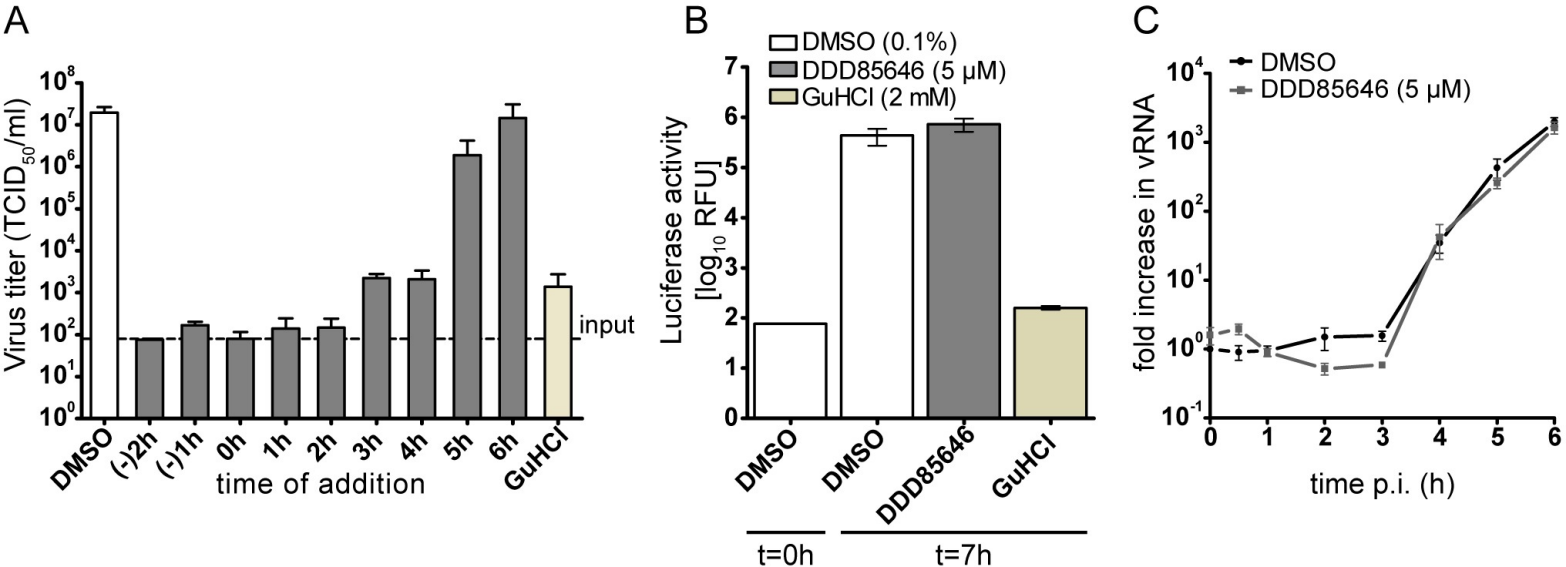
DDD85646 does not affect CVB3 replication. (A) Effect of the time-of-drug addition of DDD85646 (5 μM) on the infectious titer of progeny CVB3. The well-known inhibitor of viral replication, guanidine hydrochloride (GuHCl) was used as a control at 2 mM and DMSO served as solvent control. Each bar represents the mean ± SD, n = 3. (B) Viral RNA replication of RLuc-CVB3 in the absence (DMSO control) and presence of 5 μM DDD85646 as measured by the luciferase activity assay. GuHCl (2 mM) was used as a control inhibitor. Each bar represents the mean ± SD, n = 3. (C) The viral RNA level during a CVB3 single cycle infection was measured by RT-qPCR in the absence (DMSO control) and presence of 5 μM DDD85646 at the indicated time points and normalized to the GAPDH mRNA level. Each data point represents the mean ± SD, n = 2.

HsNMTs may exert a catalytic function in CVB3 multiplication beyond modification of the viral polyprotein. After exclusion of a direct virucidal effect of DDD85646 (S5 Fig), we performed a time-of-drug addition experiment to determine the stage of the virus life cycle at which NMT activity was of importance (Fig 4A). Addition of DDD85646 2 h prior to challenging HeLa cells with CVB3 resulted in a greatly decreased virus titer. Virus production was equally inhibited when the drug was added 2 h p.i., indicating that a severely diminished HsNMT activity had minimal influence on attachment, entry, and uncoating. The inhibitory effect was slightly reduced when DDD85646 was provided 3 h p.i., matching that of guanidine hydrochloride (GuHCl), a potent inhibitor of RNA synthesis of many picornaviruses [56], added at the beginning of infection. A modest antiviral activity was still observed when virus multiplication was allowed to proceed for 5 h before drug addition; DDD85646 became ineffective when supplied at 6 h p.i., i.e. shortly before one replication cycle has been completed. For B type coxsackieviruses, the switch from cellular to viral protein translation usually occurs 2 h p.i and viral protein synthesis levels off at about 5 h p.i. [57]. This agrees well with the period where HsNMT activity is important for virus multiplication as revealed by the time-of-drug addition experiment. We conclude that the role of HsNMT1/2 in the CVB3 infection cycle is confined to the synthetic phase, likely to maintain co-translational myristoylation of the picornaviral polyprotein.

To determine whether DDD85646 interferes with the replication of the viral genome, we used the recombinant coxsackievirus RLuc-CVB3, which expresses *Renilla* luciferase as sensitive indicator for viral RNA replication [58]. In contrast to GuHCl treated control cells, a strong luminescence was recorded at 7 h p.i., regardless of addition of DDD85646 at the time of virus challenge (Fig 4B). This indicated unperturbed viral RNA production and hence a correct processing of the non-structural precursor proteins P2 and P3. To independently corroborate the data obtained with RLuc-CVB3, we assessed replication of wt CVB3 in DDD85646-treated and control HeLa cells by RT-qPCR of newly synthesized plus-strand RNA. No significant difference in RNA replication kinetics was found in a single-cycle experiment (Fig 4C).

**Fig 5:**
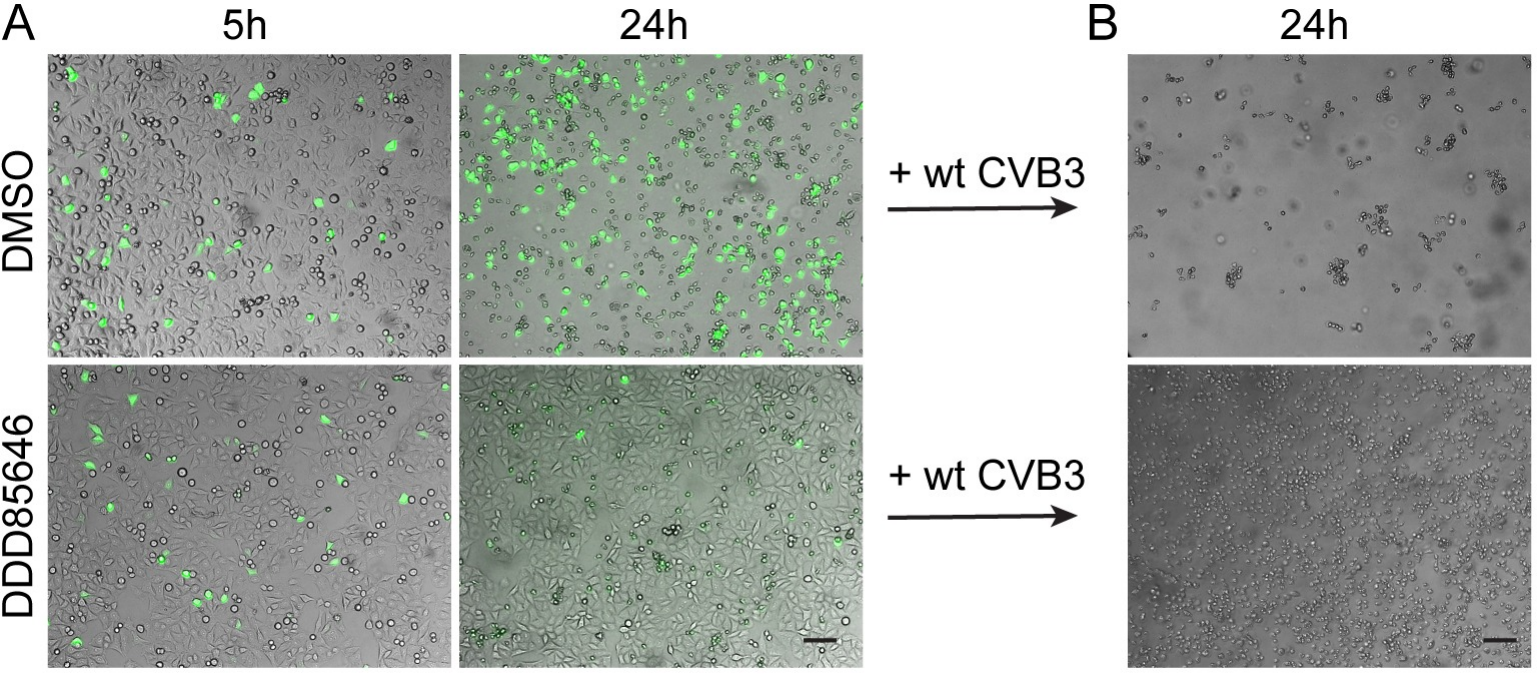
DDD85646 efficiently suppresses CVB3 cell-to-cell transmission. (A) HeLa cells were infected with CBV3-eGFP at an MOI of 0.1 in absence (DMSO control) or presence of 5 μM DDD85646 and observed under the fluorescence microscope 5 h and 24 h p.i. (B) Cells remaining intact after the 24 h multi-cycle infection with CVB3-eGFP were subsequently challenged with wt CVB3 in complete absence of the NMT inhibitor and visualized for a CPE 24 h later. Scale bar 5 μm.

From the normal viral RNA synthesis in presence of DDD85646, we infer that reduction of VP0/VP4 myristoylation blocks step(s) required for initiation of a second cycle of infection. To substantiate this reasoning, we employed recombinant CVB3-eGFP that expresses a fluorescent reporter for easy detection of infected host cells. At an MOI of 0.1, a similar small number of cells became infected at 5 h p.i. regardless of the presence of DDD85646 and, as judged from the fluorescence intensities, the viral replication rates were also similar. However, virus spread monitored at 24 h p.i. was strongly reduced in drug-treated cells when compared to the DMSO solvent control (Fig 5A). While most control HeLa cells showed a cytopathic effect (CPE), i.e. rounding and detachment from the support following the 24 h multi-cycle infection, a large number of cells were protected from CVB3-eGFP infection when DDD85646 was present. To verify that these surviving cells did not become intrinsically less supportive for virus replication as a result of an up-regulated innate immune response [59] or drug-induced changes, such as arrest in cell cycle [51, 60], we re-infected them with wt CVB3 at an MOI of 1, but this time in complete absence of the NMT inhibitor. Twenty four hours later, these cells showed massive CPE, ruling out that cell-intrinsic alterations prevented cell-to-cell transmission (Fig 5B).

### CVB3 grown in presence of DDD85646 exhibits assembly defects, accumulates provirions and has a drastically decreased specific infectivity

**Fig 6:**
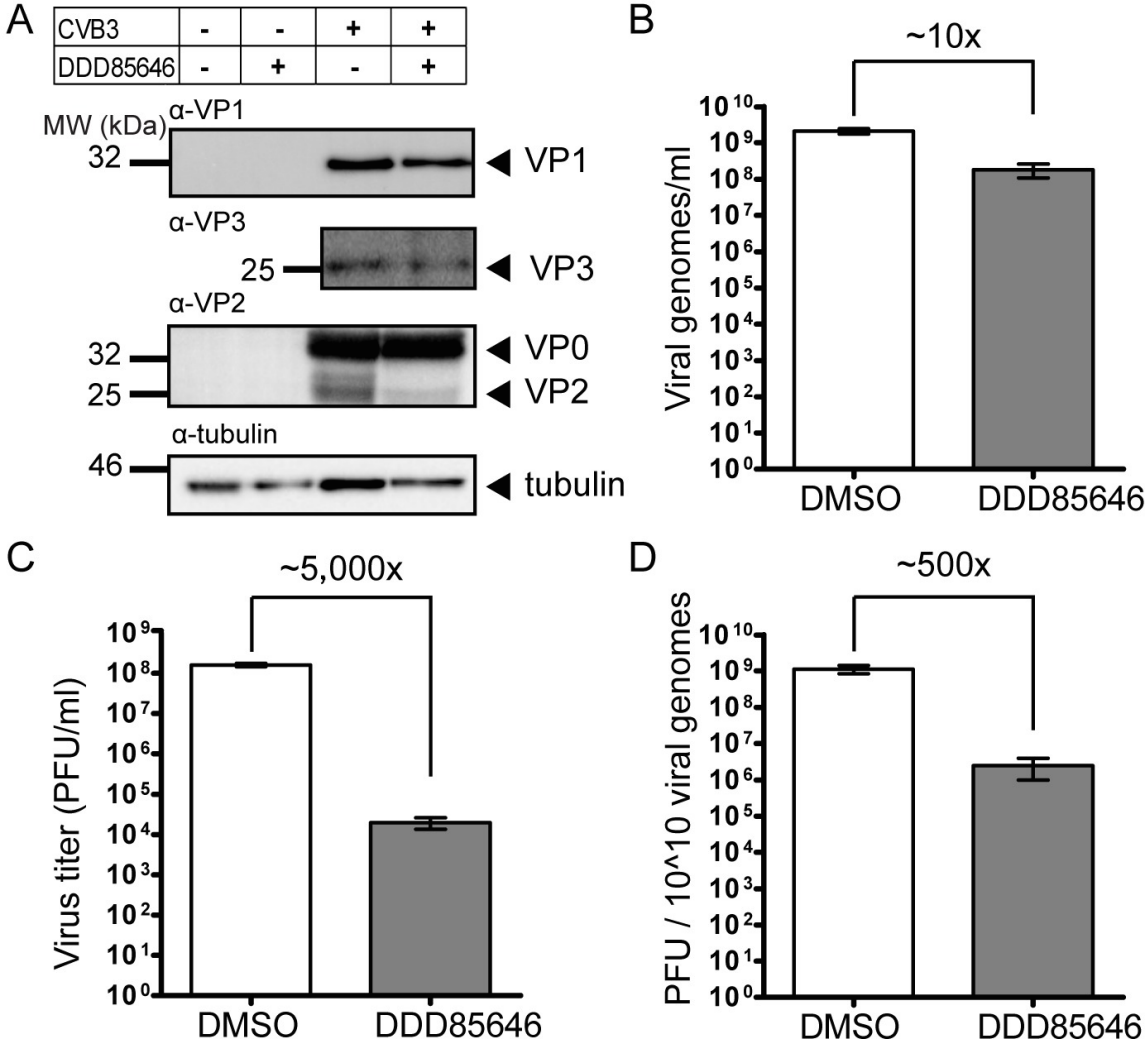
NMT inhibition by DDD85646 results in impaired CVB3 VP0 processing and drastically diminished specific infectivity. (A) Western blot analysis of VP1, VP3, VP0 and VP2 expression 6 h p.i. in lysates of Alk-12 metabolically labeled HeLa cells infected with CVB3 (MOI of 10) and treated with 5 μM DDD85646 or solvent control (DMSO). Tubulin served as a loading control. (B) HeLa cells were infected with CVB3 at an MOI of 5 in absence (DMSO control) or presence of DDD85646 (5 μM). Cell lysates were prepared at 7 h p.i. and the number of encapsidated (SuperNuclease-protected) genomes determined by RT-qPCR. Each bar represents the mean ± SD, n = 3. (C) The yield of infectious progeny virus in lysates prepared in (B) was determined by endpoint titration. The derived TCID_50_/ml values for the drug- and solvent-treated samples were multiplied by 0.7 to obtain PFU/ml. (D) Specific infectivity calculated from the data in (B) and (C) as the number of PFU per 10^10^ viral RNA genomes of progeny CVB3 propagated in the presence of DDD85646 or the solvent control (DMSO). Each bar represents the mean ± SD, n = 3.

From the above experiment, we deduce that cell-to-cell transmission of CVB3 grown in the presence of DDD85646 is impaired. This might be due to aberrant processing of the P1 precursor into capsid proteins and/or their perturbed assembly, as previously reported for myristoylation-deficient poliovirus mutants [18–24]. Western blot analysis of cell extracts from CVB3-infected HeLa cells prepared 6 h p.i. showed similar expression levels of VP1 and VP3 in absence and presence of DDD85464 (Fig 6A, two uppermost panels). Therefore, the drastic inhibition of HsNMT1/2 did not affect 3CD^pro^-mediated cleavage of the structural precursor P1. By contrast, maturation cleavage of VP0 was inefficient, as indicated by the depletion of VP2 (Fig 6A, third panel). A strong VP0 signal was also present in the lysate from CVB3-infected untreated HeLa cells, which stems largely from the substantial amounts of immature 5S protomers normally produced in infected cells. The result suggested that virus morphogenesis was hampered since in enteroviruses cleavage of VP0 is intimately linked to RNA encapsidation [6]. It was also consistent with the about tenfold lower yield (i.e. 1 log10) of encapsidated (SuperNuclease-protected) viral RNA copies recovered at 7 h p.i., measured by RT-qPCR (Fig 6B). Strikingly, the titer of the virus produced in presence of DDD85646 deceased about 5,000 fold (i.e. by 3.7 log10, Fig 6C, despite unaltered infectivity (in PFU/μg RNA) of the capsid-extracted viral genome following transfection into HeLa cells to by-pass receptor-mediated entry/uncoating steps, a method frequently employed to assess its integrity (e.g. [61, 62]) (S6 Fig). Taken together, these data document that restricting myristoylation of CVB3 resulted in about 90% less viral progeny, which had an approx. 500 fold lower specific infectivity (i.e. the ratio of infectious particles measured as PFU/ml and physical particles measured as genomes/ml) when compared to virus grown under control conditions (Fig 6D). Binding of viral progeny collected from drug-treated and DMSO-treated cells to HeLa cell was similar (S7A Fig). Monoclonal antibody-mediated blockage furthermore demonstrated virus attachment to both, coxsackie-adenovirus receptor (CAR) and decay-accelerating factor (DAF) displayed on these cells, in accordance with previous results on CVB3 variants [63–66], and no gross difference in the inhibitory pattern was observed (S7B and S7C Fig). These results attest an overall unchanged 3D structure of CVB3, when myristoylation of viral structural proteins VP0/VP4 is severely diminished, though subtle alterations of the particle inconsequential for cell attachment cannot be excluded.

**Fig 7:**
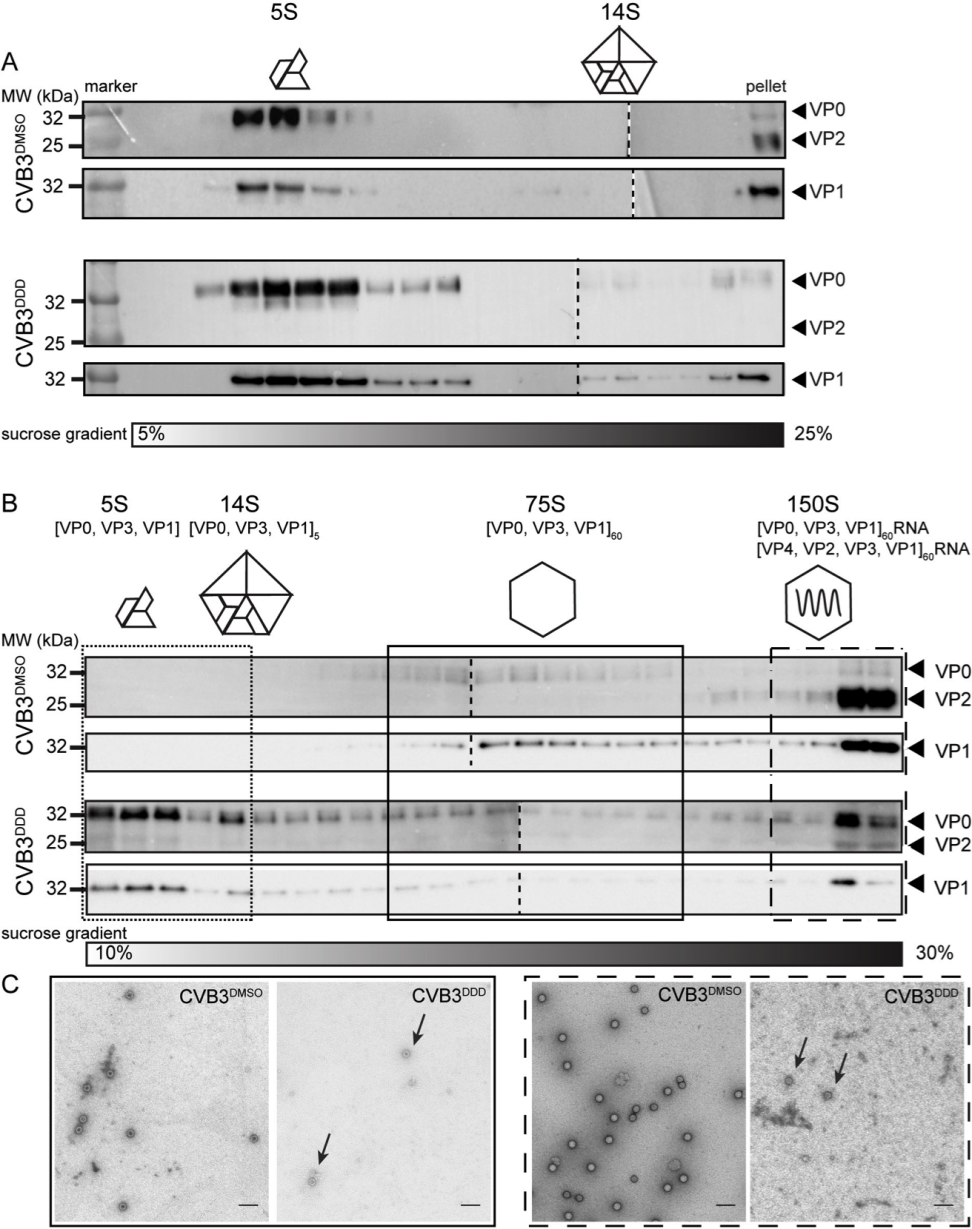
Growth of CVB3 in presence of DDD85646 results in accumulation of provirions. (A) Effect of DDD85646 on small (early) viral assembly intermediates. HeLa cells were infected with CVB3 in absence (DMSO control) or presence of the drug (5 μM) and lysates prepared 7 h p.i. were loaded onto a 5–25% (w/v) sucrose density gradient. Odd fractions taken from top to bottom were analyzed for 5S protomers and 14S pentamers by Western blotting using VP1 and VP0/VP2-specific antibodies. The resuspended pellet was similarly analyzed for the presence of large viral structures which sedimented to the bottom of the tube. Cartoon representations of the small assembly intermediates are displayed above the corresponding regions in the blot. The sucrose density is expressed as g/cm^3^. Note that images of Western blots from two different gels prepared under identical conditions were stitched together (indicated by a short-dashed line) for better appreciation of the results (B) Effect of DDD8546 on the assembly of large (late) viral assembly intermediates. HeLa cells were infected with CVB3 in absence (DMSO control) or presence of the drug (5 μM) and viral assemblies ≥14S present in lysates prepared 7 h p.i. were initially pelleted through a 30% (w/v) sucrose cushion. The respective resuspended material was loaded onto a 10–30% (w/v) sucrose density gradient for separation of 75S and 150S particles. Only 1/10 of the control sample was used to compensate the higher virus production in absence of DDD85646. Fractions were collected from top to bottom and capsid proteins visualized by Western blotting as described before. Cartoon representations of the assembly intermediates are displayed above the corresponding regions in the blot. The sucrose density is expressed as g/cm^3^. Images of Western blots from two different gels prepared under identical conditions were stitched together (indicated by a short-dashed line) for better appreciation of the results (C) Gradient fractions corresponding to 75S empty procapsids and 150S full particles (virions and provirions) were pooled separately, pelleted, and recovered particles were visualized by negative-stain transmission electron microscopy. Scale bar 100 nm.

As inhibition of HsNMTs with DDD85646 led to an about tenfold lower yield of full (i.e. CVB3 RNA-containing) virus particles, we next examined the step(s) at which virus assembly was critically blocked. Lysates of the same number of HeLa cells infected with CVB3 in presence or absence of the drug were prepared after one infection cycle (7 h p.i.) and 5S protomers and 14S pentamers fractionated on a 5–25% (w/v) sucrose density gradient. Every second fraction of the gradient was examined in a Western blot developed with VP1 and VP0/VP2 specific antibodies to reveal a possible variation in the steady-state levels of these early assembly intermediates. Control lysates exhibited a rather narrow peak of 5S protomers close to the top of the gradient and only trace amounts of 14S pentamers at steady-state. Larger virus structures were pelleted under these conditions. Based on the high proportion of VP2 over VP0 the pellet seemed to be largely composed of mature 150S virions (Fig 7A, upper two panels). Lysates from NMT inhibitor treated cells showed a considerably broader peak for the viral protomers, extending somewhat beyond the 5S value, while the level of 14S pentamers appeared just slightly elevated compared to the control lysate (Fig 7A, lower two panels). The immunoblot of the pellet comprising the large viral particles still made in presence of the NMT inhibitor showed some VP0, while VP2 was practically absent. This suggested that mostly immature viral particles such as 150S provirions and 75S procapsids had been pelleted. The amount of VP1 reporting on the total number of virus particles was furthermore reduced in the drug-exposed sample by about 10-fold in comparison to the control lysate as determined by densitometry, paralleling the decrease in genomes/ml following DDD85646-treatment (Fig 6B). Further resolution and immunoprobing of these large structures by re-centrifugation on a steeper sucrose gradient was unsuccessful due to the limiting amount of material.

Accumulation of largely immature and hence non-infectious, viral RNA-containing particles such as provirions would most probably explain the observed sharp drop in specific infectivity (Fig 6D). To substantiate the above findings, in a separate experiment we further characterized the larger CVB3 assemblies formed in presence of the NMT-inhibitor. Cell lysates were prepared 7 h p.i. from DDD85646 or DMSO-treated CVB3-infected HeLa cells. The lower amount of RNA-containing viral particles (in genomes/ml) obtained in presence of the drug was compensated for by using ten times more sample than for control conditions. Viral assemblies sedimenting at >14S were initially separated from smaller material by pelleting through a 30% (w/v) sucrose cushion. The pellet was resuspended and viral particles further resolved by ultracentrifugation in a 10–30% (w/v) sucrose density gradient. Purified RV-A2 virions (150S) and subviral particles produced by heating (80S) were used in parallel as sedimentation markers (S8 Fig). Fractions were collected from the top and probed for VP1 and VP0/VP2 by Western blotting with specific antibodies as above. As expected, the peak at 150S seen for the control CVB3 sample consisted of mostly mature virions as judged from the presence of VP1 and VP2 together with minute amounts of VP0 (Fig 7B, upper two panels). Strikingly, the 150S peak of particles made in presence of DDD85646 showed, besides VP1, a massive increase of VP0 compared to the solvent control, resulting in a VP0 to VP2 ratio of roughly 5 to 1 (Fig 7B, lower two panels). This fraction most likely comprised either a mixture of provirions with a small amount of mature virus particles or just provirions with about 20% of their VP0 cleaved into VP4 and VP2. We wish to note that others also observed co-sedimentation of enterovirus provirions and virions (at 150S), in contrast to earlier reports [67–72] and conclude that impaired myristoylation profoundly affects the cleavage of VP0 that normally converts provirions into infectious virions concomitant with RNA encapsidation.

The lack of detectable viral material in the light gradient fractions obtained from the control sample suggests that the prior centrifugation through the sucrose cushion efficiently removed 5S and 14S early assemblies (Fig 7B, upper panels). However, despite this pre-fractionation step, we still detected substantial amounts of VP0 and VP1 together with little VP2 in the top fractions of the gradient with the sample of the DDD85646-treated cells. It might indicate that these slowly sedimenting 5S/14S intermediates originate from 150S particles that disintegrated during sucrose gradient centrifugation and/or upon the initial pelleting step. Some faster moving material extending up to the 75S peak conceivably represents partially broken virus. This assumption is also in agreement with the reduced level of VP1 in the 150S peak, as compared to VP1 in the analogous peak of the DMSO control, taking into account that ten times more of the sample obtained from the infection in the presence of the drug was applied onto the gradient. Furthermore, the ratio of VP0/VP2 at the top of the gradient was distinctly higher than in the 150S peak. This led us to conclude that virus collected from drug-treated cells rather represents a mixed population mainly composed of fragile provirions [(+)ssRNA-(VP0VP3VP1)_60_] and some, more robust, virions [(+)ssRNA-(VP4VP2VP3VP1)_60_] as opposed to a single class of uniformly stable RNA-containing 150S particles with a substantial fraction of VP0 in their capsid remaining uncleaved.

Particles corresponding to the 75S and 150S peaks (Fig 7B, boxed fractions next to the respective cartoons) were further examined by negative stain transmission electron microscopy. The 75S particles of the control CVB3 (CVB3^DMSO^) as well as of the virus grown in the presence of the drug (CVB3^DDD^) appeared as hollow spheres with a dense core indicative for lack of genomic RNA and accumulation of stain, while the respective 150S nucleoprotein particles appeared as bright spheres (Fig 7C, left-hand and right-hand combined panels). Both had an average diameter of approx. 30 nm in accord with published values [15, 73]. Reflecting their lower amounts (Fig 7B), only few 75S and 150S particles were evident in electron micrographs of the sample from DDD85646-treated cells, however they were indistinguishable from their counterparts produced under control conditions.

### Inhibition of myristoylation does not alter colocalization of VP1 and 2C in the replication complex but abrogates formation of virus paracrystalline arrays

**Fig 8:**
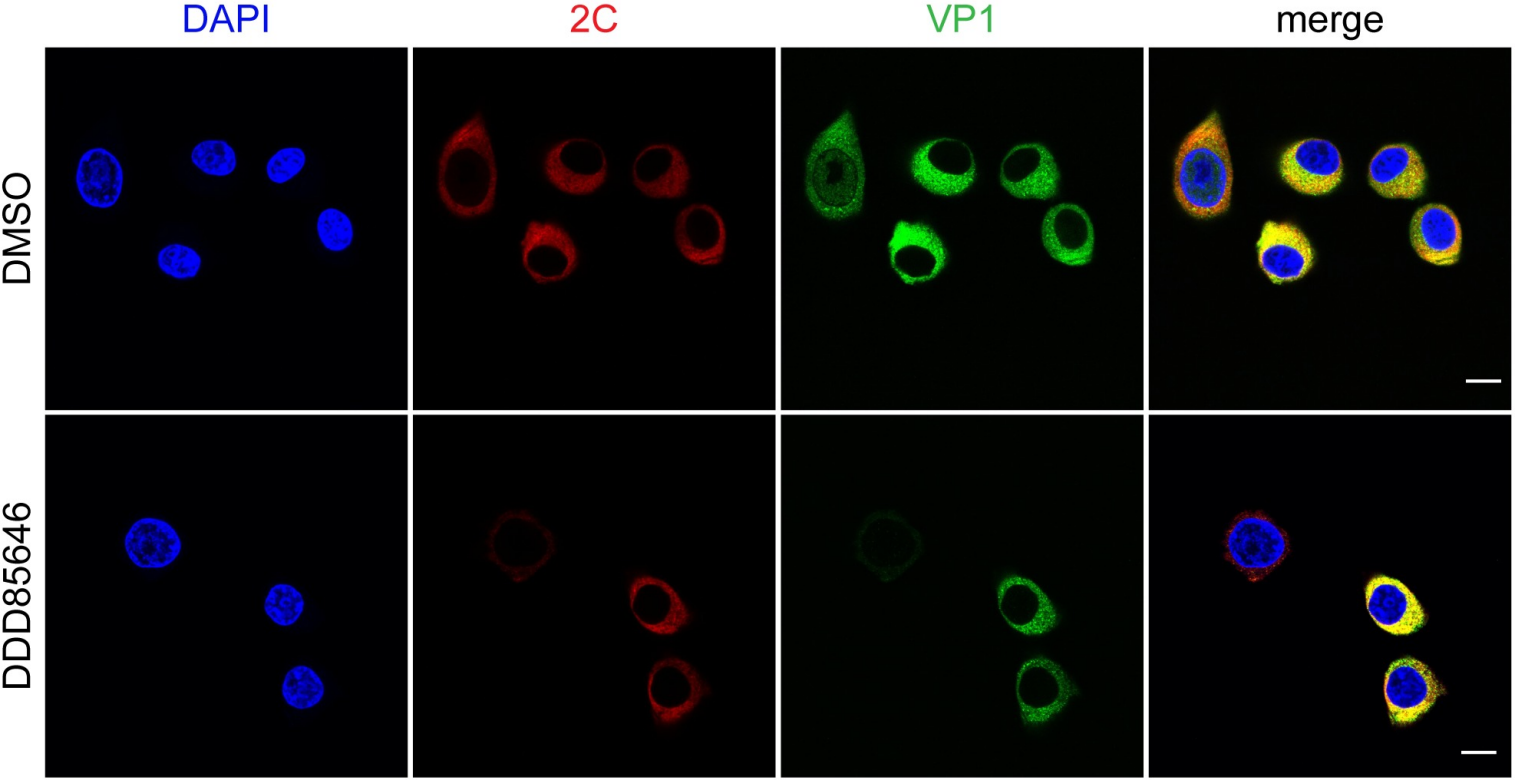
Inhibition of myristoylation by DDD85646 does not perturb CVB3 2C and VP1 colocalization. HeLa cells were infected with CVB3 (MOI of 5) in presence of 5 μM DDD85646 or DMSO (solvent control) and fixed and permeabilized with 0.5% saponin at 5 h p.i. Nuclei were visualized with DAPI (blue color). The location of VP1 was determined with a mouse monoclonal anti-enterovirus VP1 primary antibody and Alexa Fluor 488-conjugated secondary antibody (green color). 2C was probed in the same cells with an anti-CVB3 2C rabbit antibody and Alexa 555-conjugated secondary antibody (red color). Images were acquired by confocal immunofluorescence microscopy. Yellow regions in the merged images indicate colocalization. Scale bar 20 μm.

An N-linked myristoyl group, usually together with an additional targeting signal, is frequently involved in membrane attachment (as portrayed by the two-signal model; [74]). Picornavirus replication complexes (RCs), virion precursors, and perhaps newly formed full particles, localize to the surface of membranous vesicles, which are produced *de novo* [75–77]. This serves the coordination of ss(+)RNA synthesis with encapsidation [78]. According to recent findings, 14S particles, through their VP1 and/or VP3 proteins, interact with the non-structural protein 2C present in RCs. This is thought to facilitate binding of pentamers to nascent genomic RNA produced in close proximity [79]. In the context of the two-signal model, we hypothesized that the weakly hydrophobic myristate attached to VP0, by integrating into the vesicle´s lipid bilayer, might promote the interaction of intrapentameric VP1/3 with 2C. A perturbed VP0 myristoylation may lead to a decreased 2C-mediated recruitment of 14S precursors to membrane-associated RCs, resulting in inefficient virus mophogenesis. Previous studies examining the role of myristoylation in membrane targeting of polioviral precursors were rather inconclusive [80, 81]. We find by confocal immunofluorescence (IF) microscopy, that abrogation of myristoylation by DDD85646 had no significant effect on the colocalization of 2C and VP1 in perinuclear clusters of fluorescence at 5 h p.i. (Fig 8).

**Fig 9:**
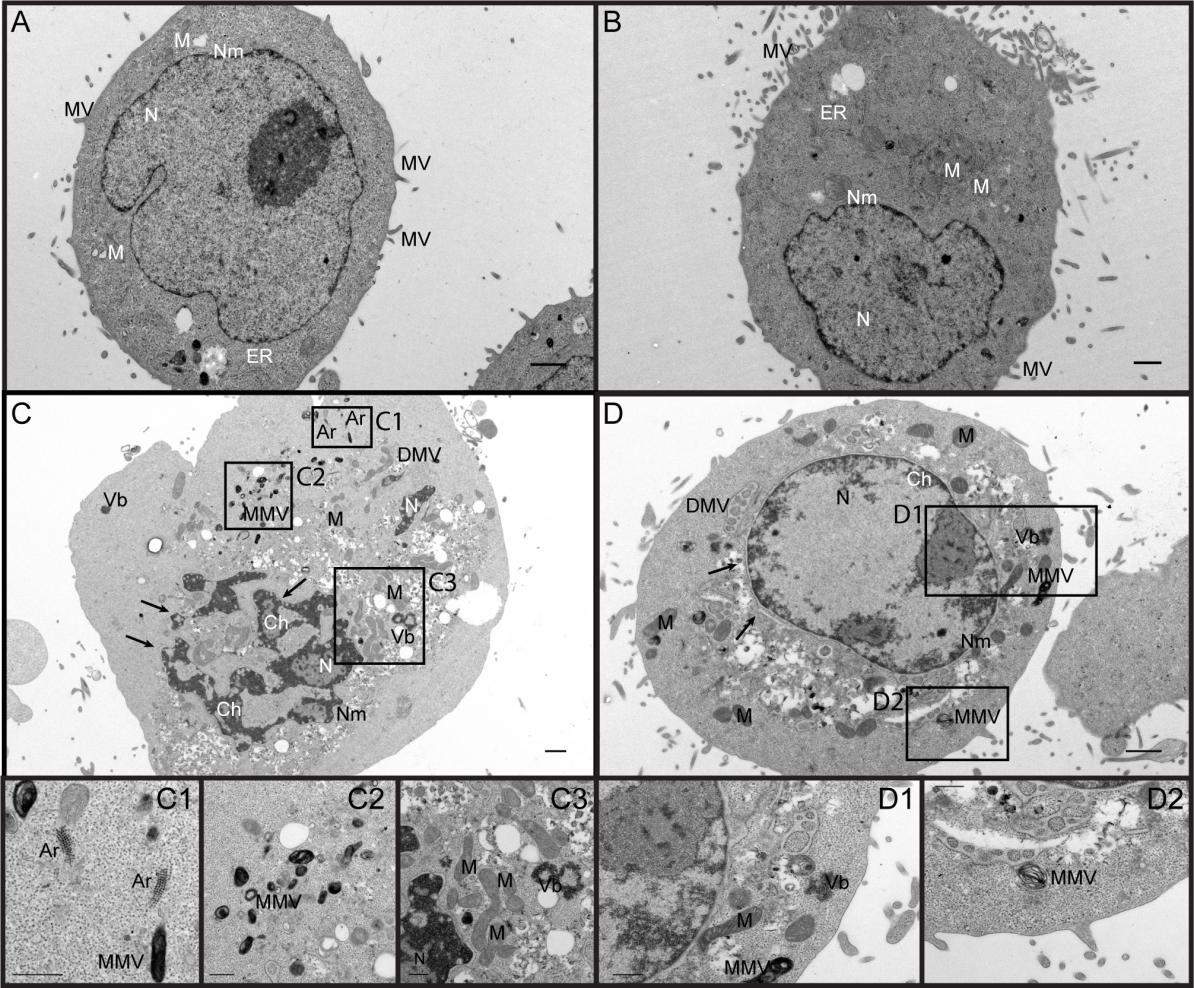
Ultrastructural changes of DDD85646- or control-treated HeLa cells infected with CVB3. (A) TEM image of a noninfected HeLa cell treated with 0.1% DMSO (solvent control). N, nucleus; Nm, nuclear membrane; M, mitochondria; ER, endoplasmic reticulum; MV, microvilli; Ch, chromatin. (B) TEM image of a noninfected HeLa cell treated with 5 μM DDD85646. (C) TEM image of a HeLa cell infected with CVB3 at an MOI of 10 in the presence of 0.1% DMSO (solvent control). The observed ultrastructural changes are typical for infection by this virus. DMV, double-membrane vesicle; MMV, multimembrane vesicle; Vb, virus bleb (an irregular virus-related aggregate); Ar, virus paracrystalline array. The black arrows indicate discontinuations of the nuclear envelope. Insets C1 to C3 are higher magnifications of the boxed regions. (D) TEM image of a HeLa cell infected with CVB3 at an MOI of 10 in the presence of 5 μM DDD85646. Virus blebs are present but no paracrystalline arrays are formed. Insets D1 and D2 are higher magnifications of the boxed regions.

A drawback of the immunofluorescence analysis is that the used enterovirus VP1-specific monoclonal antibody is unable to discriminate between intermediates and final stages of virus assembly. We hence further investigated the role of myristoylation in the morphogenesis of CVB3 at the ultrastructural level. HeLa cells were challenged with the virus in absence (DMSO control) or presence of DDD85646, incubated at 37°C for 7 h and ultrathin sections were prepared for transmission electron microscopy (TEM). One hundred micrographs for each condition were inspected for subcellular changes and formation of morphogenetic structures following infection by CVB3 as previously reported for various enteroviruses [82–85]. Non-infected HeLa cells in the absence and presence of DDD85646 displayed an organellar shape and localization typical of normal cells (Fig 9A and 9B). By contrast, CVB3-infected cells became rounded and revealed ultrastructural alterations associated with apoptosis, known to be triggered by this virus in HeLa cells [86]. The cell nucleus (N) was shrunken, the nuclear membrane (Nm) fenestrated at multiple sites, and the chromatin (Ch) condensed; most mitochondria (M) showed normal morphology in contrast to the endoplasmic reticulum (ER), which was dilated and marginalized. The cytosol was filled with numerous double-membrane vesicles (DMVs), on which RCs are anchored [87] (Fig 9C and 9D). Several multilamellar vesicles (MMV) were also apparent (Fig 9, insets C2 and D2). These virus-induced membrane structures, possibly of autophagic origin [88], closely resemble those described in a recent electron tomography study of CVB3-infected Vero E6 cells [89]; they were not markedly affected by the drug. Essentially the same number (76% and 78%) of control and drug treated cells (S9A Fig) showed about 1 to 2 irregular virus-related aggregates (virus blebs, Vb) per cell (Fig 9, insets C3 and D1). These were usually in close proximity to DMVs, contained mostly amorphous material lacking a clear border, and occasionally included round uniformly dense objects with the size of virus particles. Such aggregates likely correspond to the electron-dense material believed to represent assembly precursors and newly formed (pro)virions [84, 90]; they were unaltered in the presence of DDD85646. Remarkably, paracrystalline arrays (Ar), which often appear at this rather late stage of picornavirus infection, were detected almost exclusively in infected control cells (Fig 9C). Usually, 2–3 arrays consisting of several rows of uniformly dense particles of about 28 nm diameter were present per cell (Fig 9, inset C1). Cytoplasmic protrusions of apoptotic cells filled with elaborate particle clusters were also evident only in untreated infected cells (S9B Fig). Similar paracrystalline virus aggregates were previously reported for CVB5-infected HeLa cells [85]. They represent ordered, rather closely packed progeny virions and form when the virus attains sufficiently high intracellular concentrations. The particles in these lattice-like structures are somewhat smaller than determined by X-ray crystallography for purified virus (28 nm vs. 30 nm) due to cell shrinkage during preparation.

The above data, combined with the VP1/2C immunofluorescence colocalization study led us to conclude, that the massively reduced VP0 myristoylation does not visibly alter early precursor recruitment to still intact replication complexes. The lack of virus arrays must thus be related to a substantially diminished number of CVB3 progeny produced at these sites in presence of DDD85646, too low for their ordered intracellular aggregation, in keeping with the several-fold decrease in CVB3 particles recovered from cells lysates as determined by RT-qPCR.

## Discussion

To overcome the limitations of previous myr-deficient PV1 mutant studies, we here used gene knockout and pharmacological inhibition of cellular N-myristoyltransferases (NMTs) to explore the effect of radically diminished N-terminal myristoylation on the replication of coxsackievirus B3 (CVB3) and other representatives of the genus *Enteroviruses* and to evaluate the usefulness of NMT as a potential drug target. For the purpose of comparison we focused on CVB3, as its myristoyl group linked to VP4 displays interactions at the inner side of the capsid almost superimposable to PV1 [15]. A recent study exploring the translation initiation in CVB3 incidentally discovered an essential role for the myristoylated Gly 2 in infectivity, where its mutation to Arg resulted in abnormal polyprotein processing *in vitro*, but other possible defects have not been analyzed [91].

In mammalian cells, myristoylation is carried out by the two almost ubiquitously expressed NMT1 and NMT2 isozymes with overlapping substrate specificity, though their individual pathophysiological role is still quite elusive [11]. Using HAP1 *NMT1* and *NMT2* gene knockout clones we found that the HsNMT1 isozyme was of prime importance for CVB3 production in these cells, while gene ablation of HsNMT2 had little impact (Fig 1). A similar conclusion was very recently drawn for enterovirus 71 upon siRNA-mediated knockdown of the individual HsNMT isozymes in RD cells [40] and large-scale siRNA screens for host factors involved in HIV-1 and HBV replication also showed their critical dependence on HsNMT1 [92, 93]. A potentially differential involvement of the three HsNMT1 isoforms (NMT1L, NMT1M and NMT1S [94]) as described for HIV-1 [95] warrants further investigation.

To exclude the accidental elimination of nonenzymatic (e.g. scaffolding function(s)) by the *NMT* gene knockout, we employed DDD85646 [48] (Fig 2A) as a potent non-selective inhibitor of the human HsNMT1/2 isoforms with a high on-target mode of action in HeLa cells [47]. Orthogonal chemistry paired with in-gel fluorescence analysis demonstrated that the drug effectively decreased metabolic incorporation of the myristic acid analogue Alk-12 into VP0 in CVB3-infected cells down to at least 1% of the control (Fig 3B), being accompanied by a drastic reduction of infectious virus yield by more than 4 log10 (Fig 2E). Neither CVB3 entry, nor replication or 3C(D)^pro^-mediated processing of capsid precursor protein P1 were affected (Figs 4A–4C and 6A). Previous *in vitro* translation experiments by others reported impaired polyprotein proteolysis by 3C(D)^pro^ for PV1 and CVB3 myr-null mutants [20, 21, 91]. However, a subsequent *in vivo* analysis of myr-deficient mutant PV1 did not confirm these findings [22, 32], in line with our data on CVB3 (Fig 6A).

We found instead that CVB3 morphogenesis was clearly perturbed, as the number of RNA-filled particles in lysates of DDD85646-treated infected cells dropped by 90% relative to control values (Fig 6B). Fractionation of progeny virus and assembly intermediates on sucrose density gradients in combination with TEM confirmed the formation of a lower number of 150S particles as well as 75S empty procapsids, ultrastructurally indistinguishable from the virus control (Fig 7B and 7C). The pronounced accumulation of CVB3 5S protomers in presence of DDD85646 (Fig 7A) indicated, that the myristoyl group attached to VP0 is required for their efficient assembly into 14S precursors. However, the five-stranded β-tube built by the N-termini of VP3 packing around the icosahedral 5-fold axes, which can form only on pentamer assembly [96] apparently suffices for nucleation and/or moderate stabilization of this essential early intermediate as previously predicted [97], evidently allowing its further transition into higher order structures. Our finding agrees well with a past study by Moscufo *et al*., demonstrating a kinetic advantage of pentamer formation from myr-modified protomers over unmodified protomers made by a viable PV1 mutant with 50% reduced VP0 myristoylation [32]. Interestingly, for FMDV, either defined, but aberrantly sedimenting 17S structures or properly assembled 75S virus-like particles (VLPs, equivalent to empty procapsids) were obtained *in vitro* with recombinant protomers featuring an unmodified Gly 2 [26–28]. The reason for these disparate results has not yet been addressed.

Notably, the myristoyl group including the linked N-terminus of VP0 are disordered in the empty PV1 procapsid [96] and presumably also in the 14S precursors, with which it is in a dynamic equilibrium [6]. To what extent the stabilizing interactions of the myristate chains established in the mature virus capsid are also maintained in immature pentamers is thus elusive due to the lack of clear structural evidence. Substitution of Thr 28 in VP4 by Val to abolish its hydrogen bond with the myristoyl group from an adjacent VP4 shown in the X-ray structure of PV1 did not prevent formation of 14S particles, though assembly defects at later stages were observed [17]. Future studies are required to comprehensively determine the myristoylation-dependent interactions, which contribute to efficient protomer association and/or stability of the pentameric intermediate.

Contrary to CVB3 grown under conditions of severely inhibited VP0 myristoylation, PV1 mutants with a Gly 2 to Ala or Arg substitution shown to fully abolish this VP0 modification did not yield any 150S particles. Curiously, these mutants resulted in distinct assembly phenotypes in different labs, giving rise to just 5S particles [24], 5S and some 14S particles [22], or even 80S empty procapsids [19], possibly due to the different experimental approaches [30, 31]. However, it cannot be completely excluded, that assembly and RNA encapsidation may have also been adversely affected from substitution of the N-terminal glycine in VP0 by a side chain-bearing, less flexible amino acid residue, in addition to elimination of the myristoyl group.

Our results with the myr-depleted CVB3 are remarkably similar to those previously obtained with a PV1 mutant featuring a replacement of Ala 3 or Ser 6 by Pro in the N-terminal myristoylation motif. Despite an almost undetectable modification of VP0 [21], the level of 14S pentamers was only marginally reduced. Also, practically noninfectious virus particles sedimenting at 150S were still detected at 5- to 10-fold reduced amounts relative to wild type PV1 [22, 23]. Whether the decreased virus formation observed in these two instances in some way still depends on the very slight VP0 myristoylation is a subject for further investigations.

Recently it has been demonstrated that the non-structural protein 2C in the membrane-bound replication complex (RC) interacts with VP1 and/or VP3 possibly at the level of pentamers, thought to be crucial for progeny RNA encapsidation [79, 98]. Our confocal immunofluorescence (IF) analysis showed that the co-localization of 2C with VP1 of CVB3 in perinuclear membranes was not significantly altered in presence of DDD85646 (Fig 8), This finding rules out a prominent role of VP0 myristoylation in targeting of 14S precursors to the membrane-associated replication complex (RC), in agreement with a previous cell fractionation study examining the membrane association of wt and myr-deficient PV1 assembly intermediates [80] and is further corroborated by electron microscopy discussed below. Of note, in a very similar IF analysis, Thibaut *et al*. recently reported a disrupted co-localization of 2C with VP1 of CVB3 (Nancy) grown in glutathione (GSH) depleted cells. The protomers made under these conditions were conformationally altered and unable to assemble into pentamers [58]. Given the disparate (co)localization results, an (indirect) interaction between the myristoyl group of VP0 and GSH in stabilizing these precursors seems unlikely. Ultrastructure analysis clearly showed that dense aggregates (virus blebs) in close proximity to mostly DMVs, proposed to be the sites of progeny particle formation [75, 82, 84, 85, 99], remained unaltered compared to CVB3-infected control cells (Fig 9). Strikingly, virus paracrystalline arrays, which only form at high intracellular concentrations of progeny virus, were practically absent in infected, DDD85646-treated HeLa cells, consistent with the substantially lower number of full particles as determined by RT-qPCR. Since this became already evident *in situ* by the virtual absence of ordered viral aggregates, it strongly suggests that mechanical disruption of (pro)virions during freeze-thawing for progeny release contributed only little if at all to the drastically diminished yield.

Despite the less efficient transition of protomers made in presence of DDD85646 into 14S particles (Fig 7A), the above observations indicate that these early intermediates are not markedly depleted from the spatially still properly organized viral replication sites. This implicates a reduced flux also through the late morphogenetic pathway, most likely resulting from impaired encapsidation of the nascent (+)ssRNA by the myr-deficient 14S pentamers and/or a heightened tendency of partially formed provirions to spontaneously disassemble. In this context, the exclusive formation of completely myristoylated virions in a viable PV1 Ala 3 to Asp mutant with 50% reduced VP0 myristoylation [32] could arguably result from a profoundly higher efficacy of properly modified pentamers in condensation of the viral RNA genome. Also in accord with a defect in RNA encapsidation, unmodified protomers derived from a myr-deficient P1 precursor-expressing recombinant vaccinia virus were only inefficiently incorporated into virions of a coinfecting wild type PV1 [18].

A considerable fraction of CVB3 particles still made in presence of DDD85646 and sedimenting at 150S was in fact quite fragile and (partially) disintegrated during ultracentrifugation (Fig 7B, CVB3^DDD^). As inferred from the VP0/VP2 stoichiometry the 150S particles furthermore seemed to consist mostly of provirions. From their delicate nature they intriguingly resembled the highly unstable PV1 provirions obtained by mutation of VP2 His 195, required for the maturation cleavage of VP0 [100]. Thus, a key finding is, that the autocatalytic VP0 scission, which depends on viral RNA encapsidation and is crucial for virion stability and infectivity [71, 100, 101], appears to be critically regulated by myristoylation of VP0. We note that such hypothesis was already previously proposed by Marc *et al*. [23] based on compositional analysis of their myr-deficient PV1 mutants, and is now strongly supported by our non-mutational approach. Though quite speculative, the absence of the N-terminal C14:0 group may trigger a conformational change, which propagates along the backbone of VP0, resulting in distant structural alterations affecting the proper disposition of the maturation cleavage site (encompassing the C-terminus of VP4 and N-terminus of VP2). This would reduce but not fully prevent its scission by the nearby catalytic center comprising His 195, nucleophilic water and likely a base of the encapsidated RNA [100, 102] to account for the mature virions still produced in presence of DDD85646. A similar scenario was recently put forward to explain impaired cleavage of VP0 by mutations of Ala 107 on the outer surface of EV71 VP1 [103].

The mature CVB3 particles made in DDD85646-treated cells, if fully infectious, should theoretically suffer a just 6-fold loss in specific infectivity (the ratio of PFU to encapsidated genomes) due to their dilution by a 5-fold excess of viral RNA containing provirions, considered noninfectious [71, 101]. However, their specific infectivity instead strikingly decreased by several hundred-fold (Fig 6D) and cell-to-cell spreading was dramatically reduced compared to normal virus (Fig 5A). Thus only a very small proportion of these particles were able to productively release their genome into the cells. Their encapsidated RNA was still intact (S6 Fig) and neither attachment to the co-receptor DAF nor to CAR used for internalization [104] was substantially affected (S7A–S7C Fig). Moderate heating as an accepted surrogate for receptor-mediated uncoating of enteroviruses [105] furthermore led to complete genome release from these CVB3 particles as determined by RT-qPCR (S10 Fig). The observed block in infectivity must therefore reside in an early post-attachment step similarly as proposed by Marc *et al* [23] for their very weakly myristoylated PV1 mutants of greatly reduced infectivity. These *in vivo* results, now furnished by a virus comprising a native (non-mutated) myr-deficient VP4 can be best explained by the current model of enterovirus uncoating. It involves binding of the virion to the host cell, internalization, followed by viral receptor and/or a low endosomal pH-triggered conformational changes of the capsid proteins resulting in an expanded 135S A-particle, which possibly asymmetrically [106] externalizes the hidden VP1 N-termini and expels VP4 to form a pore for translocation of the genomic RNA into the cytosol ([107] and references therein). Formation of such an intermediate or infectosome is considered impossible when VP4 is still part of VP0 in provirions [71]. Myristoylation of recombinant VP4 furthermore noticeably facilitated pore formation in model membranes [108]. The drastically diminished specific infectivity of CVB3 (made in presence of DDD85646) is hence most likely attributable to the on average very low number of myristyolated VP4 molecules, largely insufficient for breaching the endosomal bilayer.

In the enteroviruses, the N-terminal glycine becomes accessible by removal of the initiating methionine through the action of the methionine aminopeptidase, whereas in the cardio- and aphtoviruses it is exposed by proteolytic removal of a leader peptide [6]. Irrespective of these differences, DDD85646 not only potently inhibited various members of the genus *Enterovirus* but also mengovirus, a cardiovirus. In stark contrast, HPeV-1 (a parechovirus) and AiV-1 (a kobuvirus) were practically resistant to DDD85646 treatment compared to CVB3 grown in the same cells (Table 1). At variance with most other representatives of the large *Picornaviridae* family, the VP0 proteins of parecho- and kobuviruses remain intact in the mature virions. Parechoviruses lack a consensus myristoylation signal in VP0 and are thus most likely not myristoylated [109] explaining their unresponsiveness to NMT inhibition. For HPeV-1 and 3 and Ljungan virus, numerous interactions between the RNA genome and capsid proteins VP1 and VP3 were identified in high-resolution 3D structures, suggesting a role in virion formation and stability [110–113]. These interactions not found in other picornaviruses conceivably rendered lipidation of VP0 evolutionary dispensable for assembly of parechoviruses. In kobuviruses, a large leader peptide initially attached to the P1 precursor is rapidly cleaved off by the viral protease 3C^pro^ [6], thereby exposing the N-terminal glycine of AiV-1 VP0. This glycine is part of a conserved consensus myristoylation motif (GXXX(S/T)) and was previously described to be N-terminally blocked [114], most probably by a myristate as indicated by the metabolic incorporation of the myr-analogue Az-12 into VP0 of AiV-1 infected Vero cells (S11 Fig). A 5´ terminal RNA stem-loop and the leader peptide L were found essential for AiV-1 assembly [115, 116], again indicating a possible redundancy for VP0 myristoylation in this process. Kobuviruses together with the closely related saliviruses (genus *Klassevirus*) are furthermore singled out from the other picornaviruses by having their nonstructural 3A protein N-terminally myristoylated, despite lacking a consensus myristoylation signal. Interestingly, 3A myristoylation was not essential for AiV-1 replication as was shown by site-directed mutagenesis [117]. The reason for maintenance of a myristoylated VP0 and 3A despite lacking an obvious function in infectious AiV-1 production, as inferred from its insensitivity to DDD85646, remains to be determined. Though not tested here, we would further predict that hepatitis A virus (HAV) of the genus *Hepatovirus* is also resistant to NMT inhibition, since its VP4 is non-myristoylated [118]. The strong hydrophobicity of this unusually small polypeptide compared to other picornaviruses is sufficient for endosomal pore formation [119], and 2A as (temporary) VP1 appendix appears to take over the myr-moiety´s role in virus capsid assembly [120].

Despite lacking activity against members of a few genera, our data suggest that DDD85646 has promising potential to be developed as a broad-spectrum anti-picornaviral drug. Furthermore, until now we failed to isolate stable drug-resistant CVB3 mutants in dose-escalation experiments. Drugs targeting host-factors such as NMT represent an attractive alternative to direct-acting antivirals, as the latter suffer from the rapid emergence of drug escape variants in RNA viruses, due to the high error rate of the RNA–dependent RNA polymerases [2, 121].

Taken together we have discovered that acute inhibition of NMT activity by the pan-HsNMT1/2 isozyme inhibitor DDD85646 has a potent anti-picornavirus activity with minimal cytopathic effect on infected cells over several replication cycles. With respect to CVB3, the almost complete suppression of myristoylation led to a ~10-fold reduction in virus progeny, drastic accumulation of provirons relative to virions, and a profound defect of these particles in RNA release. These seemingly disparate deficits appear to be all connected to insufficient acylation of the viral structural proteins VP0/VP4, resulting in poor viral RNA encapsidation, attenuated maturation cleavage and likely lack of functional pore formation for cytosolic transfer of the viral genome through the endosome membrane.

After finishing revision of our manuscript we realized that a paper has been very recently published by Mousnier *et al*., examining the inhibition of RV, PV and FMDV replication by a newly developed NMT inhibitor (IMP-1088) [122]. While there is a certain overlap with our data independently strengthening our conclusions, our analysis substantially extends their findings by demonstrating the pivotal role of NMT1 isozyme in this lipid modification, by showing the importance of myristoylation for the VP0 maturation cleavage and also for RNA uncoating. We furthermore report for the first time the unexpected insensitivity of two genera of picornaviruses (*Parecho-* and *Kobuviruses*) towards profound NMT inhibition.

## Methods

### Reagents

All cell culture reagents were from Gibco, except fetal calf serum (FCS), which was from Sigma-Aldrich. NP-40 and Tris were purchased from AppliChem, SDS was from Serva, D-sucrose from Roth; other chemicals were obtained from Sigma-Aldrich unless otherwise stated. DNase I, RNase A and EDTA-free complete protease inhibitor cocktail were from Roche, SuperNuclease was obtained from SinoBiological and InstantBlue from Expedon. RNase A from bovine pancreas was prepared as 100 mg/ml stock solution in 10 mM Tris-HCl, pH 7.5, 15 mM NaCl. DDD85646 (2,6-Dichloro-4-[2-(1-piperazinyl)-4-pyridinyl]-N-(1,3,5-trimethyl-1H-pyrazol-4-yl)-benzenesulfonamide, CAS No. 1215010-55-1) was synthetized as previously described ([123] and S12 Fig); it was dissolved in DMSO at 50 mM and kept in aliquots at –20°C. 2-hydroxy-myristate (2-HMA) was obtained from Enzo Life Sciences; a 10 mM stock solution was prepared in DMSO and kept at –20°C. A 1 M stock solution of guanidine hydrochloride (GuHCl) was prepared in H_2_O and kept at 4°C. Alk-12 (myristic acid alkyne, tetradec-13-ynoic acid) was from Cayman Chemical, L-azdiohomoalanine (AHA) from Invitrogen (Thermo Fisher Scientific), fatty acid free BSA was from Calbiochem, and LDS sample buffer was from Novex (Thermo Fisher Scientific). RIPA buffer (50 mM Tris-HCl pH 8.0, 500 mM NaCl, 0.1% SDS, 0.5% sodium deoxycholate, 1% NP-40, 1 mM EDTA, protease inhibitors), sodium phosphate buffer (100 mM sodium phosphate pH 7.4, 0.1% Triton-X 100, protease inhibitors), virus buffer (20 mM Tris-HCl, pH 7.5, 2 mM MgCl_2_) were made in-house and filter sterilized.

### Antibodies

The polyclonal rabbit anti-CVB3 VP3 antiserum [124] and rabbit anti-CVB3 2C antiserum [125] were provided by Karin Klingel. The mouse monoclonal anti-enterovirus VP1 antibody was from Dako (Clone 5-D8/1). The rabbit anti-CVB3 VP2 polyclonal antiserum [126] was kindly donated by Andreas Henke from the Institute of Virology and Antiviral Therapy, Medical Center at the Friedrich-Schiller-University Jena, Germany. The rabbit anti-NMT1 polyclonal antibody was purchased from Gentex and the mouse anti-NMT2 monoclonal antibody was obtained from BD Biosciences. The monoclonal mouse anti-γ-tubulin antibody was from Sigma. HRP-conjugated secondary antibodies were purchased from Jackson ImmunoResearch, and Alexa-Fluor conjugated antibodies were obtained from Thermo Fisher Scientific. For Western blot analysis all antibodies were diluted in PBSTB (0.1% Tween 20 supplemented with 2% BSA) in following dilutions: the anti-enterovirus VP1 antibody 1:1,000, anti-CVB3 VP2 1:1,000, anti-CVB3 VP3 1:500, anti-NMT1 1:5,000, anti-NMT2 1:1,000, anti-tubulin 1:5,000, HRP-secondary antibodies 1:10,000.

### Cells

All cell lines except HAP1 were originally obtained from ATCC. HeLa Ohio were maintained in minimal essential medium (MEM), HEK293 and Vero cells in DMEM, and A549 in RPMI, supplemented with 10% fetal calf serum (FCS), 1% penicillin and streptomycin and 1% L-glutamine (Pen-Strep-Glu). Caco-2 cells were grown in DMEM, 20% FCS with Glu and antibiotics. HAP1 cells were obtained from Horizon Discovery and grown in IMDM supplemented with 10% FCS and 1% Pen-Strep. HeLa-CDHR3/Y529 cells overexpressing the RV-C receptor (the cadherin-related family member 3 (CDHR3) Y529 variant) were produced and maintained as described in [127]. All cells were kept in a humidified 5% CO_2_-containing atmosphere at 37°C. In infection assays, serum concentration was reduced to 2% FCS. For infection with rhinoviruses, the infection medium was additionally supplemented with 30 mM MgCl_2_ and cells incubated at 34°C.

### Viruses and infectious clones

Rhinovirus-2 (RV-A2), rhinovirus-14 (RV-B14), coxsackievirus-B1 (CVB1), mengovirus (MV), and human parechovirus 1 (HPeV-1) were purchased from ATCC. Plasmid p53CB3/T7, containing the full-length cDNA of coxsackievirus B3 (CVB3, strain Nancy) [128] and plasmid pRLuc-53CB3/T7, which contains the Renilla luciferase gene upstream of the capsid coding region for production of a Renilla luciferase (RLuc) expressing CVB3 (RLuc-CVB3) [129] were a generous gift from Frank Kuppeveld (Institute of Veterinary Research, Faculty of Veterinary Medicine, University of Utrecht, The Netherlands). Plasmid pMKS-1-eGFP encodes an enhanced green fluorescent protein (eGFP)-expressing CVB3 (CVB3-eGFP H3 strain) [130]. The plasmid pAV-UCSF [117], carrying a full-length cDNA of Aichi virus 1 (genus *Kobuvirus*) was kindly donated by Joseph L. DeRisi (University of California San Francisco, USA). The plasmid pC15 [131], a gift from Ann Palmenberg (Institute for Molecular Virology, University of Wisconsin, USA), contains a full-length cDNA copy of the RV-C15 genome.

### *In vitro* transcription of vRNA and transfection of HEK293 cells for production of (recombinant) virus

Infectious plasmids were linearized at the 3´-end of the cDNA insert with an appropriate restriction enzyme and viral RNA (vRNA) transcribed *in vitro* using the T7 Ribomax Large Scale RNA Production System (Promega). The RNA was purified by phenol-chloroform-isoamyl alcohol extraction followed by ethanol precipitation, dissolved in RNase-free water (Quiagen) supplemented with RNasin (Promega) and stored at –80°C until further use.

HEK293 cells were transfected with *in vitro* transcribed vRNA using Lipofectamine 2000 (Invitrogen) according to the supplier´s instructions. Two days later, cells were disrupted by three freeze-thaw cycles to release the (recombinant) virus and the clarified supernatant was used for preparation of respective virus stocks in HeLa cell monolayers (HeLa-CDHR3/Y529 cells for RV-C15 and Vero cells for Aichi virus). Infectious virus titers were determined by endpoint titration expressed as 50% tissue culture infectious doses (TCID_50_) per ml [132], routinely yielding 10^7^–10^8^ TCID_50_/ml. Virus stocks were aliquoted and kept at –80°C.

### XTT cell-viability assay

Cell viability assay was essentially done as described before (Thinon *et al*. [47]). After 24, 48, and 72 h, XTT reagent (AppliChem) instead of MTT was added to the cells in the presence of phenazine methosulfate (PMS) and A_490_ and A_630_ of the colored formazan derivative reductively formed by metabolically active cells were determined 2 h later. A_490_–A_630_ was calculated for each measurement and the average value of the puromycin control subtracted from the average value of the other samples. The metabolic activity was calculated as percentage relative to the solvent control (referred as 100% viability). CC_50_ values were calculated by fitting the data to the IC_50_ function using GraphPad Prism 6.0 (GraphPad Software).

### Virus yield reduction assay

The effect of the inhibitor (DDD85646, 2-HMA, GuHCl) in a single cycle infection was determined as reduction of TCID_50_ with respect to the solvent control. Virus (CVB3, CVB1, RV-A2, RV-B14, RV-C15, Mengovirus, HPeV-1, AiV-1) was added to subconfluent cells (HeLa, A549, Caco-2, Vero) as specified in Figs 2, 4, and 6 and Table 1, grown in 12-well plates at an MOI of 1 and incubated in appropriate serum-reduced infection medium for 1 h at 37°C (34°C for rhinoviruses). Non-internalized virus was removed by washing the cells three times with PBS and fresh infection medium with inhibitor was added at concentrations specified in the corresponding figures. Infected cells were subjected to three cycles of freezing-thawing at a time p.i. appropriate for the respective virus (listed in Table 1), cellular debris was removed by low speed centrifugation and virus titers (TCID_50_) were determined as above.

### Time-of-drug addition experiments

Cells grown in 12-well tissue culture plates were infected with CVB3 at an MOI of 1 for 1 h at 37°C in infection medium and washed with PBS. DDD85646 was added to a final concentration of 5 μM at the times specified in Fig 4 starting from 2 h prior infection (–2) up to 6 h p.i. (+6) and maintained until the end of the experiment. GuHCl (2 mM), used as a control for inhibition of virus replication, was added at 1 h p.i. (+1 h). At 7 h p.i., cells were subjected to 3 freeze-thaw cycles for virus release. TCID_50_ was then determined as above.

### Renilla luciferase assay

HeLa cells grown in 12-well plates were infected with RLuc-CVB3 at an MOI of 1. Immediately after infection, DDD85646 (5 μM) or GuHCl (2 mM) was added. Addition of DMSO (0.1% final concentration) served as solvent control for the NMT inhibitor. At 7 h p.i., cells were lysed for determination of Renilla luciferase activity using the Renilla Luciferase Assay System (Promega) according to the manufacturer´s instructions. Luminescence was detected with the Synergy H1 hybrid multi-mode microplate reader (BioTek). The increase of relative light units (RLU) in the absence of GuHCl was taken as measure of RNA replication.

### Analysis of CVB3 replication in HeLa cells by real-time RT-qPCR

HeLa cells were seeded into 12-well plates to reach 80–90% confluency at the day of infection. Cells were inoculated with CVB3 at an MOI of 5 for 30 min at 4°C followed by removal of unbound virus by washing three times with PBS. Cells in one well were immediately lysed in 350 μl RLT buffer for total RNA isolation using the RNeasy Mini kit. The temperature was subsequently raised to 37°C (at 5% CO_2_) for 30 min to trigger virus internalization into the cells of the other wells. New infection medium containing either 0.1% DMSO or 5 μM DDD85646 was then added to the cells and incubation continued up to 6 h. In 1 h intervals, the medium was aspirated from a well of drug- and control-treated cells followed by a gentle wash with PBS. The cells were then directly lysed in RTL buffer as before. Lysates were frozen at –80°C until further use. Total RNA was isolated with the RNeasy Mini kit and viral genomic RNA in each sample quantified by RT-qPCR as outlined below and normalized to the mRNA level of GAPDH determined analogously (FW: 5’ gaaggtgaaggtcggagt 3’; RW: 5’ gaagatggtgatgggatttc 3’) by the ΔΔ*C*_T_ method [133].

### Determination of the CVB3 specific infectivity

HeLa cells were infected with CVB3 at an MOI of 5 for 1 h at 37°C. Unbound virus was removed by washing two times with PBS and fresh infection medium containing DDD85646 (5 μM) or DMSO (0.1%) was added. Seven h p.i. cells were subjected to 3 freeze-thaw cycles. Cellular debris was removed by low speed centrifugation and an aliquote of the supernatant was saved for virus titration. To eliminate any unpackaged viral RNA, the remaining virus-containing supernatant was treated with SuperNuclease at 25 U/ml final concentration for 30 min at 37°C. Encapsidated (SuperNuclease-protected) genomes were then isolated with Trizol LS (Invitrogen) and finally resuspended in RNase-free water. Reverse transcription (RT) of 5 μl of this solution was carried out by using Moloney Murine Leukaemia Virus Reverse Transcriptase (MMLV, Promega) according to the manufacturer’s instructions using an oligo dT primer. Quantitative real time qPCR was performed on an Eppendorf Realplex 2 Mastercycler with the Kappa Sybr Fast qPCR Master Mix (Peqlab) according to the manufacturer’s instructions. Pan entero specific oligonucleotides (FW: 5’ tcctccggcccctgaatg 3’; RW: 5’ gaaacacggacacccaaagta 3’) were used for amplification of the cDNA. The number of genomes (corresponding to the original quantity of intact, viral RNA-containing particles) was calculated from a standard curve obtained by qPCR of known amounts of plasmid p53CB3/T7. PFU/ml were calculated from the TCID_50_/ml value using the formula 1 PFU/ml = 0.7 x TCID_50_/ml; by normalization to the measured genome copy number/ml the specific infectivity is derived, expressed as the number of PFU per 10^10^ viral genomes.

### Labelling with the myristic acid analogue Alk-12 and the methionine analogue L-AHA

HeLa cells grown in 6 cm dishes until 90% confluent were incubated with medium alone or with increasing concentrations of DDD85646 (at 0.25, 0.5, 1, 5, and 10 μM) for 30 min at 37°C and 5% CO_2_. For labelling with Alk-12, the medium was replaced with fresh medium containing 0 to 10 μM of the drug and 22 μM Alk-12. For labelling with L-AHA, the medium was replaced with Met/Cys-free DMEM supplemented with 2% dialyzed FCS, 1% Pen-Strep with DDD85646 and 20 μM L-AHA. Twenty-four h later, cells were scraped off the plate, pelleted at 500 g in a benchtop centrifuge and the pellet was washed twice with ice-cold PBS. Cells were lysed in 100 μl of 100 mM sodium phosphate buffer and 25 U/ml SuperNuclease for 20 min on ice. To incorporate Alk-12 into viral proteins, HeLa cells were challenged with CVB3 at an MOI of 10 in infection medium for 1 h at 37°C. The inoculum was removed, cells washed with PBS, and incubation continued following addition of infection medium with 5 μM DDD85646 or 0.1% DMSO (control). Cells were washed with PBS at 3.5 h p.i. and labelling medium (MEM supplemented with 2% fatty acid free BSA, 1% Pen-Strep-Glu) containing DMSO or 5 μM DDD85646 was added, and incubation continued for 30 min. Alk-12 was then added to a final concentration of 22 μM. At 6 h p.i. cells were lysed as described above. Cellular debris was removed by centrifugation at 4°C and 20,000 g for 25 min in a benchtop centrifuge. Supernatants were snap frozen and kept at –80° until further use.

### CuAAC and in-gel fluorescence detection of Alk-12 and L-AHA-labelled proteins

Click chemistry grade Na-ascorbate, Tris(3-hydroxypropyltriazolylmethyl)amine (THPTA), Cu_2_SO_4_, and the fluorescent capturing reagents Cy5.5-Alkyne and 5-TAMRA-Azide were from Jena Biosciences. Na-ascorbate and Cu_2_SO_4_ were freshly prepared before each experiment as 50 mM solutions in H_2_O. Stock solutions of THPTA (10 mM in water) and capturing reagents (10 mM in DMSO) were prepared and kept in aliquots at –20°C. Cell lysates were thawed on ice. The protein concentration was determined with the BCA Protein Assay Kit (Pierce, Thermo Fisher Scientific) and adjusted to 1 mg/ml with 100 mM sodium phosphate buffer. A click mixture was prepared by adding the components from stocks in the following order with vigorous mixing after each addition; capture reagent Cy5.5-Alkyne (for L-AHA) or 5-TAMRA-Azide (for Alk-12) (1 μl, final concentration 0.1 mM), Cu_2_SO_4_ (2 μl, final concentration 1 mM), sodium ascorbate (2 μl, final concentration 1 mM), THPTA (1 μl, final concentration 0.1 mM). Six μl of a master mix was then added to 94 μl of the sample in a non-transparent microcentrifuge tube. Incubation was at 25°C with shaking at 600 rpm for 1 h. Next, 400 μl ice-cold acetone was added, the samples were quickly vortexed, kept at –20°C for 1 h and centrifuged at 14,000 g for 30 min to pellet precipitated proteins. The pellets were air-dried, 75 μl of 2% SDS in PBS, 10 mM EDTA was added and samples were vortexed. Once the proteins were completely dissolved, 25 μl of 4x LDS was added (the final concentration of proteins was ~ 1 mg/ml). The samples were incubated at 95°C for 5 min and 10 μg protein each were loaded on a 10% or 15% Tris-Tricine SDS polyacrylamide gel. After electrophoretic separation, the gel was washed three times with MilliQ water and fluorescence was recorded with a Typhoon FLA 9500 laser scanner (GE Healthcare); equal protein loading was verified by staining with InstantBlue. The same protein samples were resolved by separate SDS-PAGE, transferred to an Immobilon-P PVDF membrane (EDM Millipore) and probed with an anti-enterovirus VP1, anti-CVB3 VP2, anti-CVB3 VP3, and anti-γ-tubulin antibody (as loading control) as specified further below.

### Sucrose gradient sedimentation

HeLa cells seeded in one 15 cm culture plate at ~80% confluency were infected with CVB3 at an MOI of 10. After 1 h adsorption, unbound virus was washed away and infection medium containing DMSO or 5 μM DDD85646 added. Seven h p.i. cells were collected with trypsin, washed 2x with PBS and lysed in 500 μl of TNE buffer (10 mM Tris-HCl pH 7.4, 100 mM NaCl, 1 mM EDTA, 0,5% NP-40, 1x protease inhibitor stock) for 20 min on ice. Cellular debris was removed by low speed centrifugation, and the resulting supernatant fractionated through a 5–25% (w/v) sucrose density gradient (prepared in TNE buffer) by centrifugation for 16.5 h at 202,048 g in a SW40 Ti rotor (Beckman) at 4°C (zero break). Fractions (~400 μl) were collected from the top of the gradient and the pellets obtained from this centrifugation were each resuspended in 400 μl TNE buffer. 10 μl of each odd fraction inclusively the dissolved pellet were subject to SDS-PAGE, the proteins transferred to an Immobilon-P PVDF membrane and analyzed as described below.

For separation of larger viral assemblies, a 10–30% (w/v) sucrose density gradient was used. HeLa cells grown in twenty 15 cm culture plates were infected with CVB3 at MOI of 5. Following 1 h adsorption, DMSO or DDD85646 (5 μM) was added to the infection medium. Seven h p.i. cells were disrupted by 3 cycles of freezing and thawing, clarified supernatants combined and viral material was pelleted in a SW32 Ti rotor (Beckman) through a 30% (w/v) sucrose cushion prepared in virus buffer for 3.5 h at 118,018 g and 10°C. Pellets were resuspended in virus buffer containing DNase I (Roche) and RNase A (Roche) at a final concentration of 5 μg/ml each, and incubated for 15 min at RT. N-laurlysarcosine was added to a final concentration of 1% (w/v). The samples were kept overnight at 4°C and insoluble material was removed by a 15 min centrifugation at 20,000 g, 4°C. Virus from DDD85646-treated cells (CVB3^DDD^) and virus from DMSO treated cells (CVB3^DMSO^; 1/10 to compensate for the different yield) diluted to equal volumes of 0.5 ml with virus buffer was each overlaid onto 5 ml of a 10–30% (w/v) sucrose gradient made in virus buffer and centrifuged for 30 min in a SW55 Ti rotor (Beckman) at 286,794 g and 4°C. Two hundred μl aliquots were collected from top to bottom and frozen at –80°C until further use. Ten μl of each fraction were run on a 15% Tris-Tricine SDS polyacrylamide gel and proteins transferred to an Immobilion-P membrane for Western blot analysis. Viral proteins were detected using anti-CVB3 VP2 or anti-enterovirus VP1 specific antibodies as described below. Fractions containing empty or full particles were pooled separately and viral material was pelleted in a TLA100.3 rotor (Beckman) at 207,995 g for 1 h at 4°C. The pellets were resuspended in 30 μl of virus buffer and stored at –80°C until further use.

### Western blot analysis

Whole cell extracts were prepared by lysis of cells in RIPA buffer for 30 min on ice and clarified at 20,000 g for 5 min at 4°C. Equal amounts of total protein (10 to 30 μg) determined with the BCA Assay Kit were separated on a 10% or 15% Tris-Tricine SDS polyacrylamide gel and transferred to an Immobilion-P PVDF membrane using the Trans-Blot Turbo system (Bio-Rad). The membrane was blocked for 1 h at RT in PBSTB and incubated with primary antibody overnight at 4°C. After washing, the membrane was incubated with HRP-conjugated secondary antibody for 1 h at RT. Bands were revealed by chemiluminescence (SuperSignal West Pico from Pierce, Thermo Fisher Scientific) and images were captured with a Chemidoc system (Bio-Rad). The band intensity was quantified using the ImageLab Software (Biorad). Expression of γ-tubulin as loading control was determined by stripping the membrane in glycine buffer (2.5 mM glycine, 1% SDS, pH 2.0) for 1 h at RT, followed by rinsing with PBST, re-blocking in PBSTB and probing with a mouse monoclonal anti-γ-tubulin antibody according to the procedure described above.

### Confocal microscopy

For VP1 and 2C co-localization studies, HeLa cells were grown until subconfluent on a 12 mm coverslip (Marienfeld) placed in the well of a 24-well plate. The medium was replaced by infection medium and CVB3 was added at an MOI of 5. After 1 h at 37°C, virus inoculum was removed and replaced with fresh medium containing 5 μM DDD85646. DMSO (0.1%) was used as solvent control. Five h p.i. cells were washed with PBS, fixed with 4% PFA, and permeabilized with PBS containing 0.5% saponin. After blocking in 0.5% fish gelatin in PBS for 15 min, cells were incubated with a combination of anti-enterovirus VP1 antibody and anti-CVB3 2C—both diluted 1:200 in blocking solution—for 1 h. Excess antibody was removed by washing with PBS, followed by a 1 h incubation of the cells with appropriate Alexa-Fluor conjugated secondary antibody diluted 1:1,000 in blocking buffer. Cell nuclei were counterstained with DAPI. All steps were carried out at room temperature. The samples were examined in a LSM 700 confocal laser scanning microscope (Zeiss), and images were processed with Image*J*.

### Imaging of infected cells and virus spread

For analysis of virus spread, HeLa cells were grown in 12-well plates and infected with CVB3-eGFP at an MOI of 0.1 in infection medium for 1 h at 37°C and 5% CO_2_. Unbound virus was removed by washing several times with PBS, fresh infection medium containing 5 μM DDD85646 or 0.1% DMSO (solvent control) was added and incubation continued. Five and 24 h p.i. cells were examined using an Axio Observer.Z1 epifluorescent microscope. The medium of the surviving cells was replaced with fresh medium without drug, followed by infection with wt CVB3 at an MOI of 1 and analysis for a cytopathic effect (CPE) 24 h p.i. by bright field microscopy.

### Transmission electron microscopy

For transmission electron microscopy (TEM) analysis of purified CVB3, a small aliquot (3–5 μl) of virus was adsorbed for 1 min to the backside of a carbon-coated 400 mesh copper hexagonal grid that had been glow-discharged just before use. Excess sample solution was blotted away with a filter paper without letting the sample dry. Five μl of 2% uranyl acetate in water was applied to the grid and blotted off immediately. The staining was repeated for 1 min. After a final blotting of excess solution, the grid was air-dried and examined with an FEI Morgagni 268D (FEI) operated at 80 kV. Images were acquired using an 11 megapixel Morada CCD camera (Olympus-SIS). In our hands, a concentration of ~ 1x10^9^ virus particles/ml was normally required for proper visualization. For TEM of infected cells, 3x10^6^ HeLa cells were seeded into 6 cm tissue culture dishes and inoculated with CVB3 in infection medium at an MOI of 10. After adsorption for 1 h at 37°C, cells were rinsed with PBS and fresh medium was added containing 5 μM DDD85646 or 0.1% DMSO (solvent control) and incubation was continued at 5% CO_2_ and 37°C. Uninfected HeLa cells were treated identically. At 7 h p.i. cells were scraped from the dish, transferred to a benchtop centrifuge tube and fixed with 2.5% glutaraldehyde in 0.1 M sodium phosphate buffer, pH 7.2 overnight at 4°C. Cells were pelleted at 500 g for 5 min at RT, washed once with PBS, post-fixed in sodium phosphate-buffered 1% osmium tetroxide, dehydrated in a graded series of acetone, and embedded in Agar 100 resin (Agar Scientific). Seventy nm sections were cut with a diamond knife (Diatome) and post-stained with 2% uranyl acetate and Reynolds lead citrate. Sections were examined with the FEI Morgagni as above.

### Quantification and statistical analysis

All experiments were done at least in duplicate for a total of ≥ 2 biological replicates. Data are displayed as means ± standard deviation (SD). Statistical significance was determined using the unpaired two-tailed Student’s t test. The actual p value and sample size n of each experimental group are provided in the respective figure legends.

## Supporting information

S1 Appendix Supplemental materials and methods. (DOCX)

S1 Fig Characterization of HAP1 KO cells and rescue of CVB3 production by transfection with HsNMT1 cDNA. (A) Phase contrast microscopy of HAP1 wt, NMT1^KO^, and NMT2^KO^ single KO cells; 100x magnification. (B) Western blot analysis of NMT isozyme expression in parental HAP1 cells and single NMT KO cells probed with antibodies against NMT1, NMT2, and γ-tubulin (loading control). (C) HAP1 cells (wt, NMT1^KO^) were transfected with pmCherry-C1 plasmid or co-transfected with pmCherry-C1 and an HsNMT1 expressing plasmid (SC113026) by electroporation. Fluorescence analysis shows mCherry production in a large fraction of electroporated cells. Images are representative of two independent transfections. (D) About 20 h post transfection the HAP1 wt and NMT1^KO^ cells were inoculated with CVB3 at an MOI of 1 and infectious virus titers were measured 7 h p.i. Each bar represents the mean ± SD, n = 3.
(TIF)

S2 Fig Supplementation of MA rescues infectious titers of CVB3 grown in presence of 2-HMA but not DDD85646. (A) HeLa cells were infected with CVB3 at an MOI of 1, treated with 5 μM DDD85646 or (B) 20 μM 2-HMA in absence or in presence of increasing concentrations of myristic acid (MA; 0.5–100 μM) and progeny virus in cell lysates prepared 7 h p.i. was titrated as TCID_50_/ml. Each bar represents the mean ± SD, n = 3.
(TIF)

S3 Fig NMT inhibition in various cell lines results in a similar concentration-dependent cytotoxicity. DDD85646 was added for 24 h to the medium of HeLa, Caco2, Vero, and A549 cells at concentrations indicated and cell viability was determined with the XTT assay. Each data point represents the mean ± SD, n = 9.
(TIF)

S4 Fig DDD85646 inhibits Alk-12 incorporation into cellular and viral proteins but does not affect host cell translation. (A) The myristic acid analogue Alk-12 was added to cultivated HeLa cells in the presence of increasing concentrations of DDD85646 as indicated. After 24 h cells were lysed and Alk-12 labelled proteins ligated to 5-TAMRA-azide via the click reaction. Total cellular protein was separated by SDS-PAGE and 5-TAMRA-tagged polypeptides revealed by in-gel fluorescence. The structure of the myristic acid analogue (Alk-12) is shown on top of the gel; InstantBlue staining of the same gel verifies equal loading. (B) HeLa ells were incubated with the methionine analog L-azidohomoalanine (AHA) in the presence of increasing concentrations of DDD85646. Metabolically labelled proteins were processed and detected as in (A) except for using Cy5.5-alkyne in the click-reaction. The structure of the methionine analog (AHA) is shown on top of the gel; InstantBlue staining of the same gel verifies equal loading. (C) Uncropped version of the in-gel fluorescence image shown in Fig 3B. Note that the band expected for the small myristoylated VP4 (derived by maturation cleavage of VP0) is completely obscured by by-products of the click reaction as mentioned in the main text.
(TIF)

S5 Fig DDD85646 has no direct virucidal activity on CVB3. CVB3 was treated with 5 μM DDD86646 or DMSO (as solvent control) for 2 h at 37°C and the mixtures used to infect HeLa cells (corresponding to an MOI of 5 before treatment). Following attachment, drug and unbound virus were removed by washing cells 3 times with PBS; seven h p.i. progeny virus was released by three freeze-thaw cycles and infectious titer was assessed by endpoint dilution as TCID_50_/ml. Bars represent the mean ± SD for each condition, n = 3.
(TIF)

S6 Fig Transfection of capsid-extracted viral RNA. HeLa cells were transfected with equal amounts of viral genomic RNA extracted from purified CVB3^DDD^ and CVB3^DMSO^ particles obtained by propagation of CVB3 in HeLa Ohio in presence of 5 μM DDD85646 or DMSO (solvent control). Cell lysates prepared 60 h post transfection were used to determine virus yield by end point dilution as the 50% tissue culture infective dose (TCID_50_) per ml. Shown on the y-axis of the bar plot is the specific infectivity obtained for CVB3^DMSO^ and CVB3^DDD^ RNA, calculated from the data as the number of PFU (= TCID_50_ x 0.7) per μg transfected viral RNA genomes.
(TIF)

S7 Fig CVB3 produced in presence of DDD85646 has no appreciable defect in binding to DAF and CAR. (A) Equal amounts of CVB3^DDD^ and CVB3^DMSO^ (obtained by propagation of CVB3 in HeLa cells in presence of 5 μM DDD85646 or DMSO as solvent control) quantified by RT-qPCR as SuperNuclease protected genomes (corresponding to an MOI of 1 for CVB3^DMSO^) were added to HeLa cells grown in 24-well plates and allowed to attach for 1 h at 4°C. Cells were washed with PBS and the amount of cell-associated viral RNA genomes was measured by RT-qPCR and normalized to the analogously determined quantity of GAPDH mRNA. Each bar represents the mean ± SD, n = 4. (B) HeLa cells were preincubated for 1 h at 4°C with anti-CAR monoclonal antibody (clone RmcB), anti-DAF monoclonal antibody (clone BRIC 216), anti-CAR + anti-DAF monoclonal antibodies, or mouse IgG1 isotype-control monoclonal antibody (each at 10 μg/ml), or left untreated. Cells were rinsed with PBS and equal amounts in genomes/cell of CVB3^DMSO^ or (C) CVB3^DDD^ (corresponding to an MOI of 1 for the former) were added and incubation continued for 1 h at 4°C. Cells were washed with PBS and cell-associated viral RNA genomes were measured and normalized to GAPDH mRNA as in (A). Data are displayed as percent binding of CVB3^DDD^ or CVB3^DMSO^ in presence of the antibodies relative to the untreated control set to 100%. Each bar represents the mean ± SD, n = 4.
(TIF)

S8 Fig Sucrose gradient calibration by sedimentation of RV-A2 full and empty particles. Highly pure samples of RV-A2 150S native (full) and 80S (empty) subviral particles (devoid of RNA and VP4, obtained by heating of native virus for 30 min at 55°C) were each sedimented through a 10–30% (w/v) sucrose density gradient. Fractions (200 μl) taken from top to bottom were analyzed for the presence of full 150S (top panel) and empty 80S particles (bottom panel) by Western blotting using the VP2/VP0-specific monoclonal antibody 8F5. The presence of a weak VP0 band in both blots results from the small amounts of 150S provirions in the native virus preparation, which also transform into 80S particles upon heating of the sample by expelling their genomic RNA (but not VP4 being part of VP0). The sucrose density expressed as g/cm^3^ is depicted at the bottom. In each instance images from two individual Western blots have been stitched together (indicated by the short-dashed line) for better appreciation of the results. The numbers between the two blots indicate the fractions (1, top to 25, bottom) examined for the sedimentation range of RV-A2 80S or 150S particles (displayed as cartoons and corresponding composition above their respective peak fractions). The corresponding upper and lower bound fractions were used to define the equivalent sedimentation range for the 75S and 150S particles of CVB3^DDD^ and CVB3^DMSO^, centrifuged under identical conditions and similarly revealed by immunoblotting with anti-VP2/VP0 and VP1 specific antibodies (Fig 7B).
(TIF)

S9 Fig Percentage of CBV3-infected HeLa cells with virus blebs and apoptotic cell protrusion displaying virus arrays. (A) Bar**-**graphs displaying the fraction of cells, which exhibit no or one to several virus blebs in thin sections of HeLa cells at 7 h p.i. upon challenge with CVB3 in presence of 5 μM DDD85646 or the DMSO solvent control. (B) TEM image showing a fragmented apoptotic CVB3-infected cell (MOI of 10) exhibiting multiple cytoplasmic protrusions filled with arrayed virus particles.
(TIF)

S10 Fig Heat-induced uncoating of CVB3. Aliquots (100 μl) of CVB3^DDD^ or CVB3^DMSO^ at 10^9^ genomes/ml were incubated at 37, 41, 45, 49, and 56°C for 30 min to thermally trigger viral RNA uncoating. The samples were incubated with SuperNuclease to digest the released genomes; the remaining capsid-protected viral genomes were extracted with TRIzol and quantified by RT-qPCR. The percentage was plotted against the incubation temperature (°C) with 100% assigned to the value obtained for the 37°C control sample. CVB3^DMSO^ particles most extensively uncoat between 45–49°C, while about 80% of the CVB3^DDD^ particles (likely the fragile provirions) release their RNA genome already between 37–41°C; the remaining 20% (likely mostly the about fivefold under-represented, more stable myr-deficient virions) uncoat over a similar temperature window as CVB3^DMSO^.
(TIF)

S11 Fig In-gel fluorescence detection of azidomyristate (Az-12) labeled Aichi virus 1 VP0. (A) Vero cells were either sham-infected or infected with AiV-1 at an MOI of 10. The myristic acid analogue Az-12 was added 4 h p.i. and incubation continued for 6 h to allow metabolic incorporation into viral proteins. Cells were lysed 10 h p.i., Az-12-bearing proteins were ligated to the fluorescent reporter Cy5.5-alkyne by click chemistry, separated by 15% SDS-PAGE and visualized via in-gel fluorescence. This revealed a band at ~ 40 kDa uniquely present in the extract from virus-infected cells (right lane compared to the non-infected control (left lane)), close to the M_r_ of 38.9 kDa predicted for VP0 of AiV-1. (B) A Coomassie blue-stained band at the same molecular weight corresponding to VP0 is present in a highly purified Aichi virus 1 sample run in parallel.
(TIF)

S12 Fig ^1^H and ^13^C NMR spectra and chemical shifts of the NMT inhibitor DDD85646 and its immediate precursor. (PDF)
