# Supplementary material for: Cellular N-myristoyltransferases play a crucial picornavirus genus-specific role in viral assembly, virion maturation, and infectivity
Source: PLoS Pathog. 2018 Aug 6;14(8):e1007203. doi: 10.1371/journal.ppat.1007203 (PMC6089459; doi:10.1371/journal.ppat.1007203)
Supplement: S1 Appendix — (DOCX) [file ppat.1007203.s001.docx]

**Supplemental material and methods**

**Transient transfection of HAP1 cells followed by infection with CVB3 Nancy**

A plasmid containing the (untagged) full-length human NMT1 (HsNMT1) cDNA sequence (NCBI RefSeq NM_021079) under the control of a CMV promoter/enhancer (TrueClone SC113026) was purchased from OriGene. The pmCherry-C1 expression vector (Clontech) expressing the mCherry fluorescent protein was used to determine transfection efficiency. HAP1 wt and HAP1 NMT1 KO cells were each transfected with pmCherry-C1 (5 µg) or co-transfected with pmCherry-C1 (5 µg) and SC113026 (5 µg) by electroporation with the Neon Transfection System (Thermo Fisher Scientific). Cells (1 x 10^6^) were resuspended in Electrolytic Buffer E2 (Thermo Fisher Scientific) and electroporated at 1350 V with one pulse of 35 ms using 100 µl Neon Tips. Cells were subsequently seeded into 6-well plates and 3 ml of IMDM supplemented with 20% FBS without antibiotics was added to each well and replaced with antibiotics-containing medium after 4 h incubation at 37°C, 5% CO_2_. Twenty h post transfection, expression of mCherry was monitored with a Zeiss Axio Observer Z1 (Carl Zeiss) and the percentage of fluorescent cells was determined from images taken at 10 different positions of the well using Image*J*. Cells in one well of a 6-well plate of each transfection experiment were then harvested for quantification, to allow proper MOI adjustment. Cells in the remaining wells were washed with PBS, CVB3 Nancy in infection medium (IMDM supplemented with 2% FBS) was added at an MOI of 1 and cells were incubated with gentle agitation for 30 min at 37 °C and 5% CO_2_. Unbound virus was removed by washing with PBS and wells were replenished with infection medium and further incubated at 37°C, 5% CO_2_. 7 h post infection (one cycle of infection), cells in each well were scraped off and lysed by three cycles of freeze and thaw followed by centrifugation at 15,000 g for 10 min, 4°C. The supernatant was used for determination of the infectious titer expressed as TCID_50_ by endpoint dilution.

**Virucidal activity assay**

Hela cells were cultivated in 12-well plates in MEM, 10% FCS supplemented with 1% Pen-Strep and glutamine (complete medium) to reach 80% confluency at the day of the experiment. CVB3 was exposed to 5 μM DDD85646 or 0.1% DMSO (as solvent control) in infection medium (MEM, 2% FCS, 1% Pen-Strep and glutamine) for 2 h at 37 °C and was then used to infect the monolayers (corresponding to an MOI of 5 before treatment). Non-attached virus and drug were removed by washing cells 3 times with PBS, progeny virus was released 7 h p.i. by three freeze-thaw cycles, and infectious titer was assessed by endpoint dilution on HeLa cells as TCID_50_/ml.

**Determination of the specific infectivity of viral genomic RNA extracted from CVB3 capsids**

HeLa Ohio cells were infected with CVB3 Nancy at an MOI of 1 in the presence of 5 µM DDD85646 or 0.1% DMSO (solvent control). Progeny virus (CVB3^DDD^ and CVB3^DMSO^) was recovered 7 h p.i. by freezing and thawing and cellular debris were removed by centrifugation at 15,000 g for 10 min at 4 °C. Supernatants were then treated with SuperNuclease (SinoBiological, 25 U/ml final concentration) for 30 min at 37 °C, viral particles were pelleted by ultracentrifugation at 120,000 g for 3 h at 4°C and resuspended in 400 µl of 10 mM Tris-HCl (pH 7.4) and 1 mM MgCl_2_. The genomic RNA was extracted from the capsids with TRIzol LS (Invitrogen, Thermo Fisher Scientific) – chloroform, ethanol precipitated and dissolved in RNase-free water. HeLa Ohio cells were seeded into 24-well plates to reach 80% confluency at the time of transfection. For each viral RNA sample, 0.1 µg were diluted in Opti-MEM (Gibco, Thermo Fisher Scientific), combined with 4 µl Lipofectamine 2000 diluted in Opti-MEM and the RNA-Lipofectamine mix added to the cells. After 60 h incubation at 37 °C, 5% CO_2_, the cytopathic effect was well developed and the cells were subjected to three cycles of freeze and thaw followed by centrifugation at 15,000 g for 10 min at 4 °C to remove cellular debris. Progeny virus titer in the supernatants (expressed as TCID_50_/ml) was determined by endpoint dilution. The obtained values were converted to PFU/ml by multiplying by 0.7 in order to calculate the specific infectivity in PFU/μg of transfected (capsid-extracted) viral RNA.

# Quantification of CVB3 cell-binding and antibody-mediated blockage by RT-qPCR

HeLa cells were seeded into a 24-well plate at 2 x 10^5^ cells/well in complete medium to reach 80% confluency at the day of the assay. The monolayers were washed with PBS and preincubated for 1 h at 4 °C with anti-DAF MAb (clone BRIC 216; Santa Cruz Biotechnology) and/or anti-CAR MAb (clone RmcB; Millipore), previously shown to block binding of CVB3 to the respective receptors DAF and CAR [1, 2]. IgG1 from murine myeloma (Sigma-Aldrich) was added separately as isotype-matched control. Each antibody was used at a final concentration of 10 µg/ml in 200 μl MEM, 1% FCS. Cells incubated with plain medium served as no-treatment control. Unbound antibody was subsequently removed by washing with PBS and cells were either incubated for 1 h at 4 °C with CVB3^DMSO^ at an MOI of 1 or the equivalent amount of CVB3^DDD^ in genomes/cell according to prior qPCR quantification (primers FW: 5’ tcctccggcccctgaatg 3’ and RW: 5’ gaaacacggacacccaaagta 3’) of reverse transcribed viral RNA extracted from a small aliquot of both virus preparations obtained as described above. Cells were then washed three times with cold PBS and the total RNA, including any cell-associated viral RNA, was extracted with TRIzol LS - chloroform. Viral genome copy number, corresponding to the originally attached virus, was quantified by RT-qPCR as before and normalized to the level of GAPDH mRNA determined analogously (with primer pairs FW: 5’ gaaggtgaaggtcggagt 3’ and RW: 5’ gaagatggtgatgggatttc 3’).

**Thermal-induced RNA release**

Aliquots (100 µl) of CVB3^DDD^ and CVB3^DMSO^ at 10^9^ genomes/ml (quantified as above; corresponding to approx. 10^8^ PFU/ml for the latter) diluted in PBS pH 7.4 were heated at 37, 41, 45, 49, and 56 °C for 30 min and then immediately placed on ice. Any released genomes were subsequently digested with SuperNuclease (25 U/ml) for 30 min at 37 °C. Viral RNA protected within the remaining native capsids was extracted with Trizol LS - chloroform, ethanol-precipitated, and the quantity of genomes for each condition was determined by RT-qPCR as above. A melting temperature (at which 50% of purified virus particles release their RNA) of ~ 45 °C was reported previously for CVB3 ([3]).

**Sucrose gradient calibration with full and empty RV-A2 particles as sedimentation markers**

One hundred µg of RV-A2 purified as described [4] (consisting of native virions and small amounts of provirions which both sediment at 150S) were diluted in 0.5 ml 50 mM Tris-HCl pH 7.5, 25 mM NaCl and heated for 30 min at 55 °C. This results in almost complete release (heat-induced uncoating) of the viral RNA genome, together with most VP4, from the native virions, thereby converting to 80S subviral particles [5]. The heated virus sample and the same amount of a native (non-heated) RV-A2 were separately overlaid on 5 ml of a 10-30% (w/v) sucrose gradient prepared in virus buffer and centrifuged for 30 min in a SW55Ti rotor (Beckman) at 55,000 rpm and 4 °C. Two hundred μl aliquots were then collected from the top of the gradient; 20 μl of each fraction were separated on a 15% Tris-Tricine SDS polyacrylamide gel and proteins transferred to an Immobilion-P PVDF membrane for Western blot analysis. The presence of viral proteins VP2 (Mr 29.0 kDa; native 150S virion and 80S subviral particle) and VP0 (Mr 36.3 kDa; 150S provirion, 80S heated (empty) provirion capsid) was revealed using the RV-A2 VP2/VP0 specific mouse monoclonal antibody 8F5 [6] (1 mg/ml stock diluted 1:1000 in PBSTB). For each peak (empty 80S particles and full 150S particles) an upper and lower bound fraction was determined by visual inspection of the band intensities of the individual blots and used to identify the corresponding sedimentation range for the analogous CVB3 particles in the Western blots of Fig 7B.

**In-gel fluorescence analysis of Az-12 metabolically labeled VP0 of Aichi virus 1**

Vero cells were challenged with AiV-1 at MOI of 10 for 1 h at 37 °C; unbound virus was removed by washing with PBS and fresh infection medium (DMEM, 2% FCS, 1% Pen-Strep and glutamine) added. Sham-infected cells were used as a control. Three h p.i. the monolayers were rinsed with PBS and incubated with labeling medium (DMEM supplemented with 2% fatty acid free BSA, 1% Pen-Strep, 1% L-Glu) for 30 min. The myr-analogue Az-12 (12-Azidododecanoic acid, C_12_H_23_N_3_O_2_; Thermo Fisher Scientific) was then added to a final concentration of 10 μM followed by 6 h incubation at 37 °C to allow metabolic incorporation into aichiviral proteins predicted to be naturally myristoylated (Az-12 is practically equivalent to the myr analogue Alk-12, which was used for labeling CVB3 VP0 (e.g. [7])). Ten h p.i. (i.e. after one AiV-1 replication cycle), cells were rinsed with PBS, harvested and pelleted by centrifugation at 500 g for 5 minutes. The pellets were washed twice in PBS and lysed in ice-cold 100 mM sodium phosphate pH 7.4, 0.1% Triton-X 100 supplemented with protease inhibitors and SuperNuclease (25 U/ml) for 20 min on ice. Cellular debris was removed by centrifugation at 20,000 g for 20 minutes at 4 ºC. One hundred μg of total protein per sample was subsequently used in a Cu^I^-catalyzed click reaction with the Cy5.5-alkyne fluorophore (Jena Bioscience), followed by separation on a 15% Tris-Tricine SDS polyacrylamide gel and visualization of myr-analogue-labeled proteins by their in-gel fluorescence on a Typhoon FLA 9500 (GE Healthcare). A purified Aichi virus 1 sample (kindly donated by Pavel Plevka, Laboratory of Structural Virology, Central European Institute of Technology, Masaryk University, Brno, Czech Republic [8]) was electrophoretically separated in parallel and stained with Coomassie Brillant Blue for visualization of the individual capsid proteins VP0, VP3 and VP1.

**Supporting references**

1. Hsu KH, Lonberg-Holm K, Alstein B, Crowell RL. A monoclonal antibody specific for the cellular receptor for the group B coxsackieviruses. Journal of virology. 1988;62(5):1647-52. PubMed PMID: 2451756; PubMed Central PMCID: PMCPMC253193.

2. Martino TA, Petric M, Brown M, Aitken K, Gauntt CJ, Richardson CD, et al. Cardiovirulent coxsackieviruses and the decay-accelerating factor (CD55) receptor. Virology. 1998;244(2):302-14. doi: 10.1006/viro.1998.9122. PubMed PMID: 9601501.

3. Martikainen M, Salorinne K, Lahtinen T, Malola S, Permi P, Hakkinen H, et al. Hydrophobic pocket targeting probes for enteroviruses. Nanoscale. 2015;7(41):17457-67. doi: 10.1039/c5nr04139b. PubMed PMID: 26440968.

4. Weiss VU, Subirats X, Kumar M, Harutyunyan S, Gosler I, Kowalski H, et al. Capillary electrophoresis, gas-phase electrophoretic mobility molecular analysis, and electron microscopy: effective tools for quality assessment and basic rhinovirus research. Methods Mol Biol. 2015;1221:101-28. doi: 10.1007/978-1-4939-1571-2_9. PubMed PMID: 25261310.

5. Hewat EA, Blaas D. Cryoelectron microscopy analysis of the structural changes associated with human rhinovirus type 14 uncoating. Journal of virology. 2004;78(6):2935-42. PubMed PMID: 14990711; PubMed Central PMCID: PMCPMC353739.

6. Skern T, Neubauer C, Frasel L, Grundler P, Sommergruber W, Zorn M, et al. A neutralizing epitope on human rhinovirus type 2 includes amino acid residues between 153 and 164 of virus capsid protein VP2. J Gen Virol. 1987;68 ( Pt 2):315-23. doi: 10.1099/0022-1317-68-2-315. PubMed PMID: 2434607.

7. Charron G, Zhang MM, Yount JS, Wilson J, Raghavan AS, Shamir E, et al. Robust fluorescent detection of protein fatty-acylation with chemical reporters. J Am Chem Soc. 2009;131(13):4967-75. doi: 10.1021/ja810122f. PubMed PMID: 19281244.

8. Sabin C, Fuzik T, Skubnik K, Palkova L, Lindberg AM, Plevka P. Structure of Aichi virus 1 and its empty particle: clues towards kobuvirus genome release mechanism. Journal of virology. 2016. doi: 10.1128/JVI.01601-16. PubMed PMID: 27681122; PubMed Central PMCID: PMCPMC5110158.
