# Supplementary figures and images for: Cellular N-myristoyltransferases play a crucial picornavirus genus-specific role in viral assembly, virion maturation, and infectivity
Source: PLoS Pathog. 2018 Aug 6;14(8):e1007203. doi: 10.1371/journal.ppat.1007203 (PMC6089459; doi:10.1371/journal.ppat.1007203)

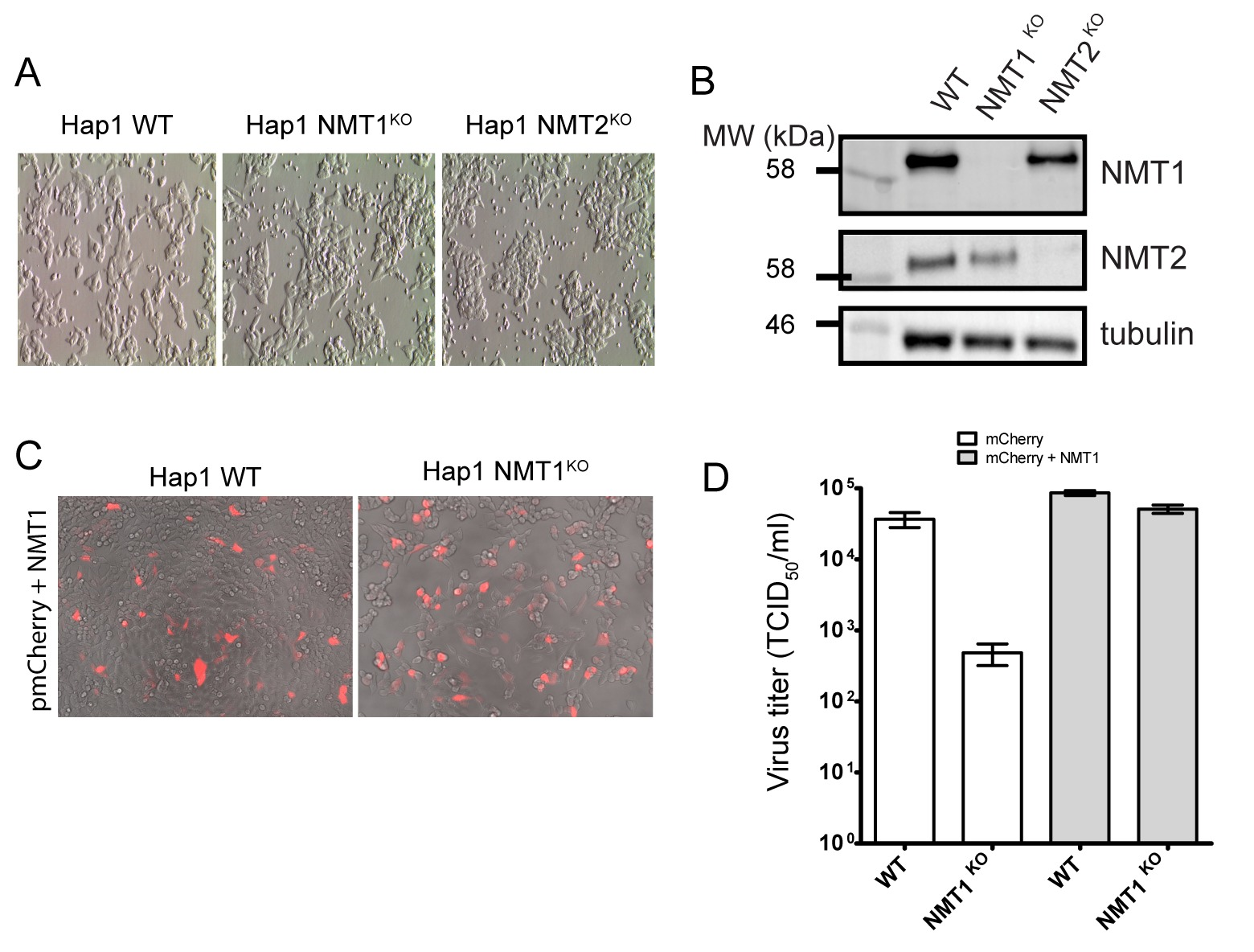

Supplement: S1 Fig — (A) Phase contrast microscopy of HAP1 wt, NMT1KO, and NMT2KO single KO cells; 100x magnification. (B) Western blot analysis of NMT isozyme expression in parental HAP1 cells and single NMT KO cells probed with antibodies against NMT1, NMT2, and γ-tubulin (loading control). (C) HAP1 cells (wt, NMT1KO) were transfected with pmCherry-C1 plasmid or co-transfected with pmCherry-C1 and an HsNMT1 expressing plasmid (SC113026) by electroporation. Fluorescence analysis shows mCherry production in a large fraction of electroporated cells. Images are representative of two independent transfections. (D) About 20 h post transfection the HAP1 wt and NMT1KO cells were inoculated with CVB3 at an MOI of 1 and infectious virus titers were measured 7 h p.i. Each bar represents the mean ± SD, n = 3. (TIF) [file ppat.1007203.s002.tif]

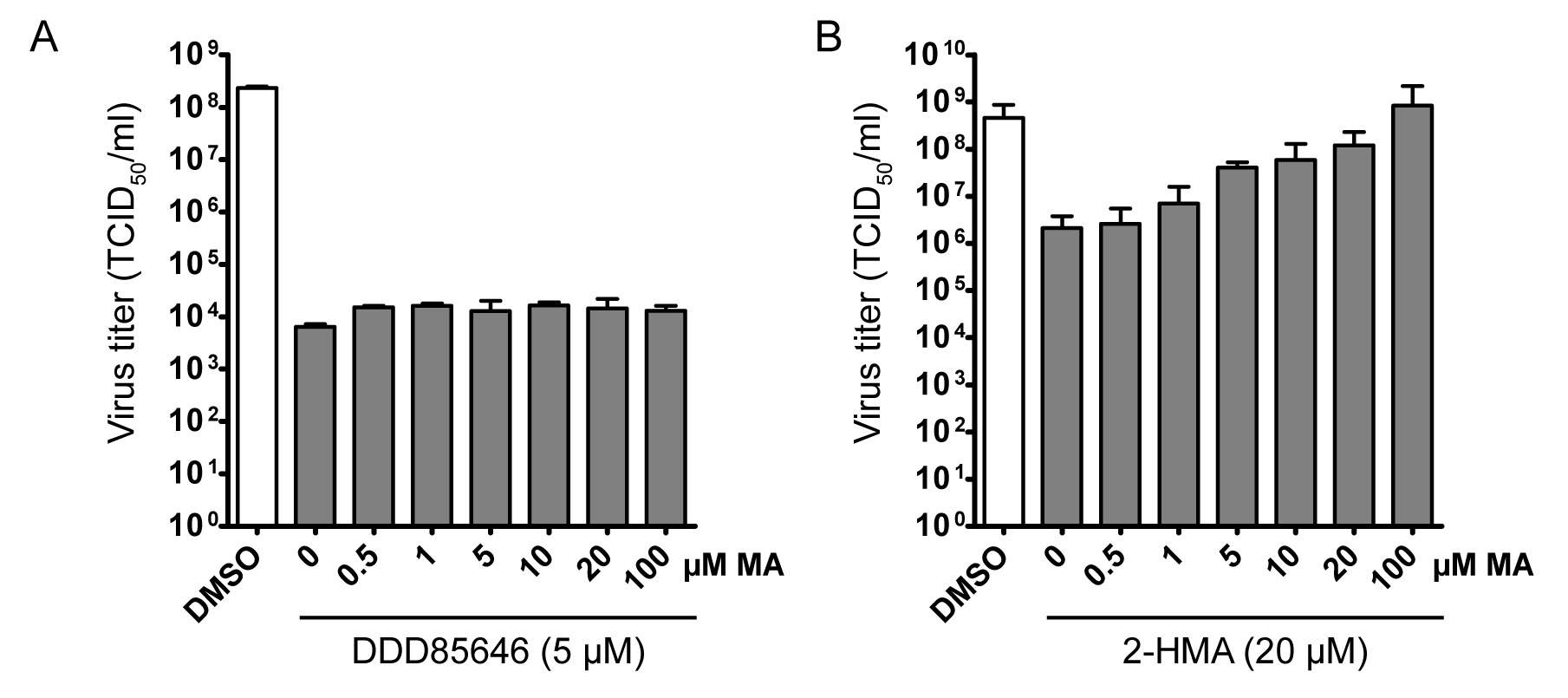

Supplement: S2 Fig — (A) HeLa cells were infected with CVB3 at an MOI of 1, treated with 5 μM DDD85646 or (B) 20 μM 2-HMA in absence or in presence of increasing concentrations of myristic acid (MA; 0.5–100 μM) and progeny virus in cell lysates prepared 7 h p.i. was titrated as TCID50/ml. Each bar represents the mean ± SD, n = 3. (TIF) [file ppat.1007203.s003.tif]

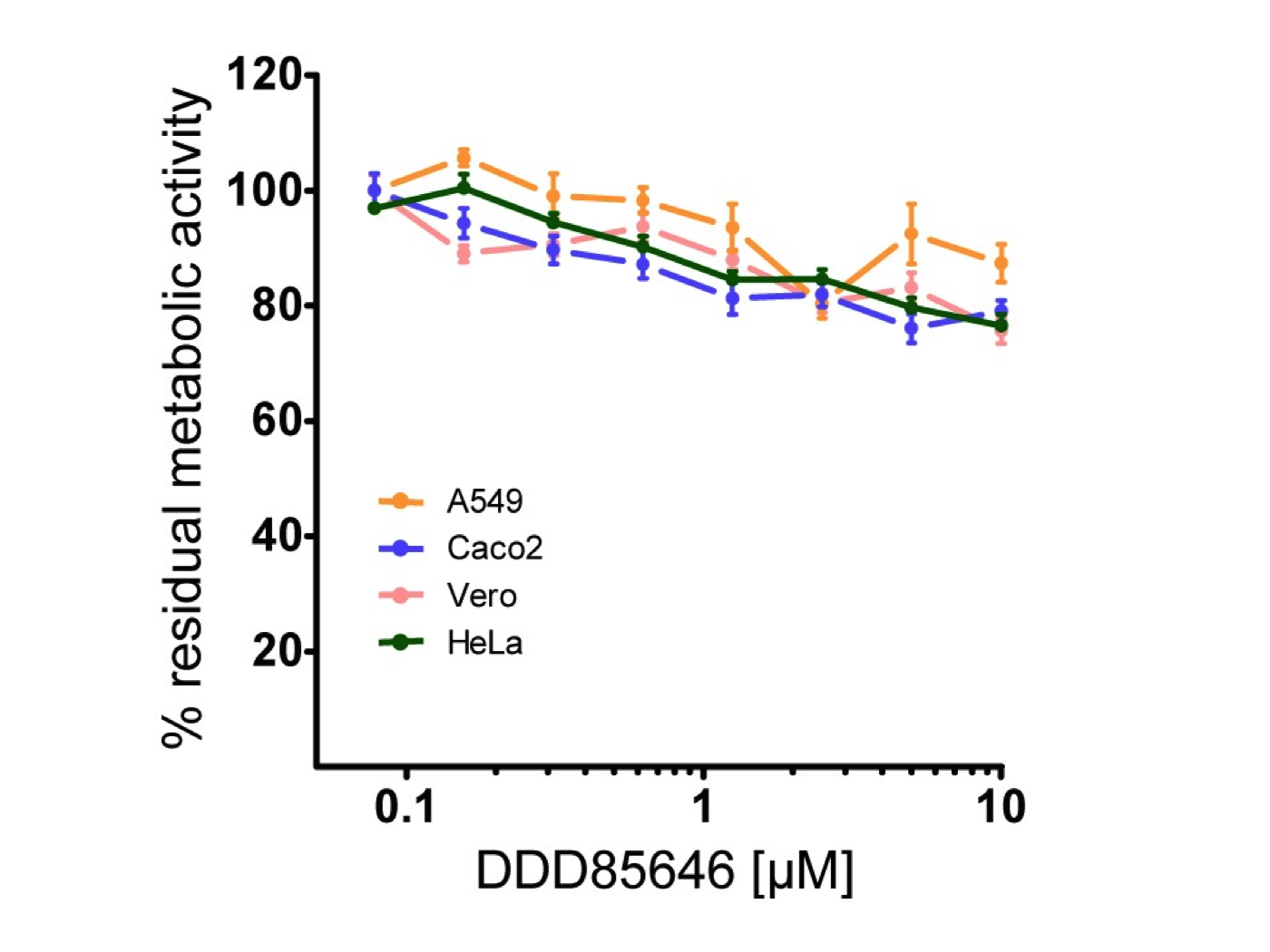

Supplement: S3 Fig — DDD85646 was added for 24 h to the medium of HeLa, Caco2, Vero, and A549 cells at concentrations indicated and cell viability was determined with the XTT assay. Each data point represents the mean ± SD, n = 9. (TIF) [file ppat.1007203.s004.tif]

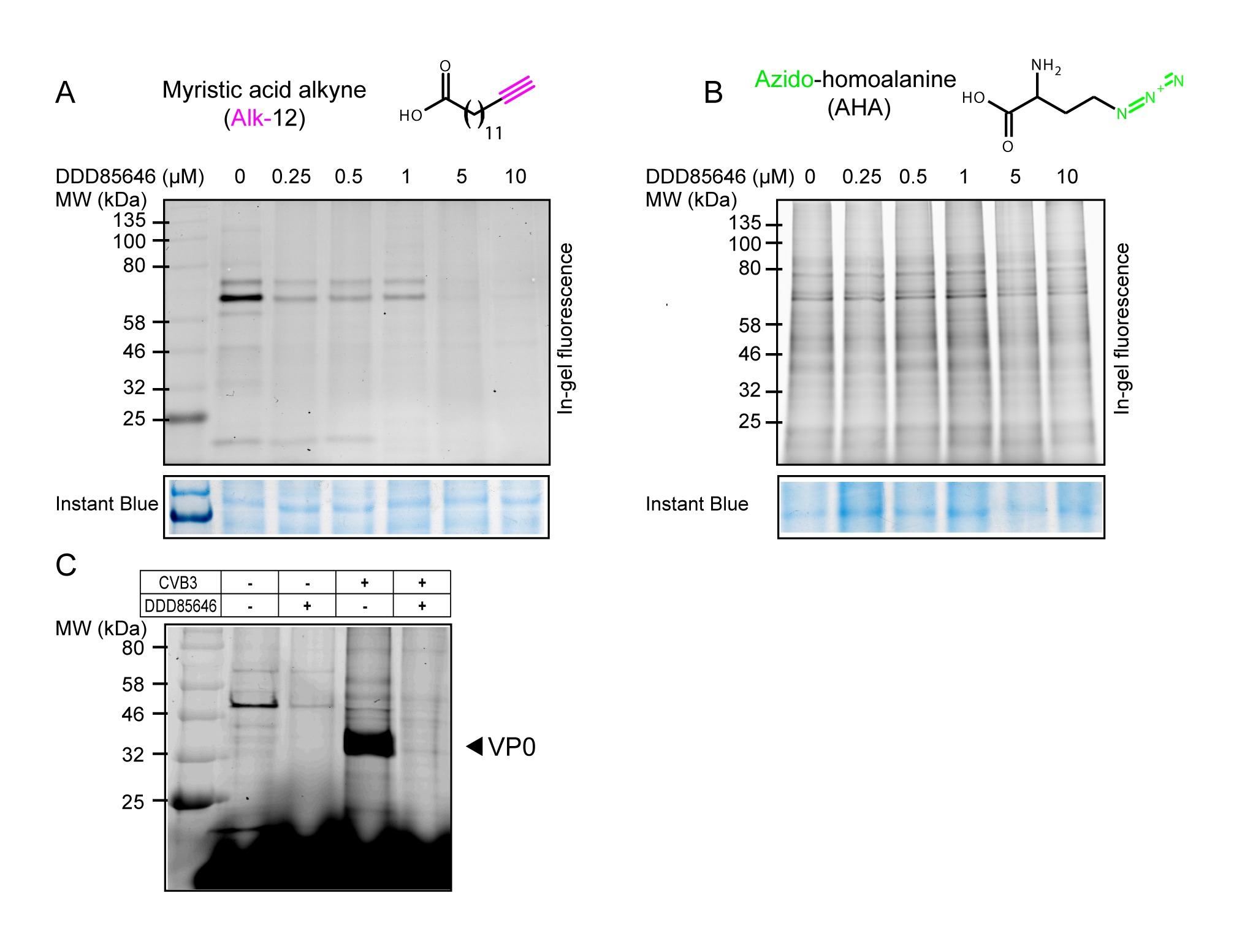

Supplement: S4 Fig — (A) The myristic acid analogue Alk-12 was added to cultivated HeLa cells in the presence of increasing concentrations of DDD85646 as indicated. After 24 h cells were lysed and Alk-12 labelled proteins ligated to 5-TAMRA-azide via the click reaction. Total cellular protein was separated by SDS-PAGE and 5-TAMRA-tagged polypeptides revealed by in-gel fluorescence. The structure of the myristic acid analogue (Alk-12) is shown on top of the gel; InstantBlue staining of the same gel verifies equal loading. (B) HeLa ells were incubated with the methionine analog L-azidohomoalanine (AHA) in the presence of increasing concentrations of DDD85646. Metabolically labelled proteins were processed and detected as in (A) except for using Cy5.5-alkyne in the click-reaction. The structure of the methionine analog (AHA) is shown on top of the gel; InstantBlue staining of the same gel verifies equal loading. (C) Uncropped version of the in-gel fluorescence image shown in Fig 3B. Note that the band expected for the small myristoylated VP4 (derived by maturation cleavage of VP0) is completely obscured by by-products of the click reaction as mentioned in the main text. (TIF) [file ppat.1007203.s005.tif]

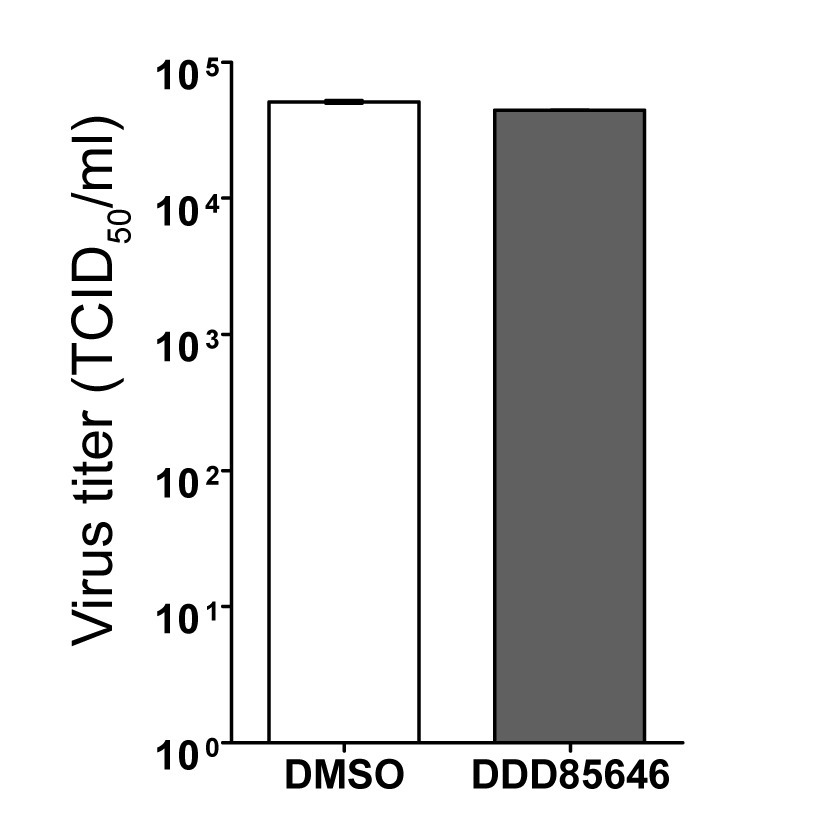

Supplement: S5 Fig — CVB3 was treated with 5 μM DDD86646 or DMSO (as solvent control) for 2 h at 37°C and the mixtures used to infect HeLa cells (corresponding to an MOI of 5 before treatment). Following attachment, drug and unbound virus were removed by washing cells 3 times with PBS; seven h p.i. progeny virus was released by three freeze-thaw cycles and infectious titer was assessed by endpoint dilution as TCID50/ml. Bars represent the mean ± SD for each condition, n = 3. (TIF) [file ppat.1007203.s006.tif]

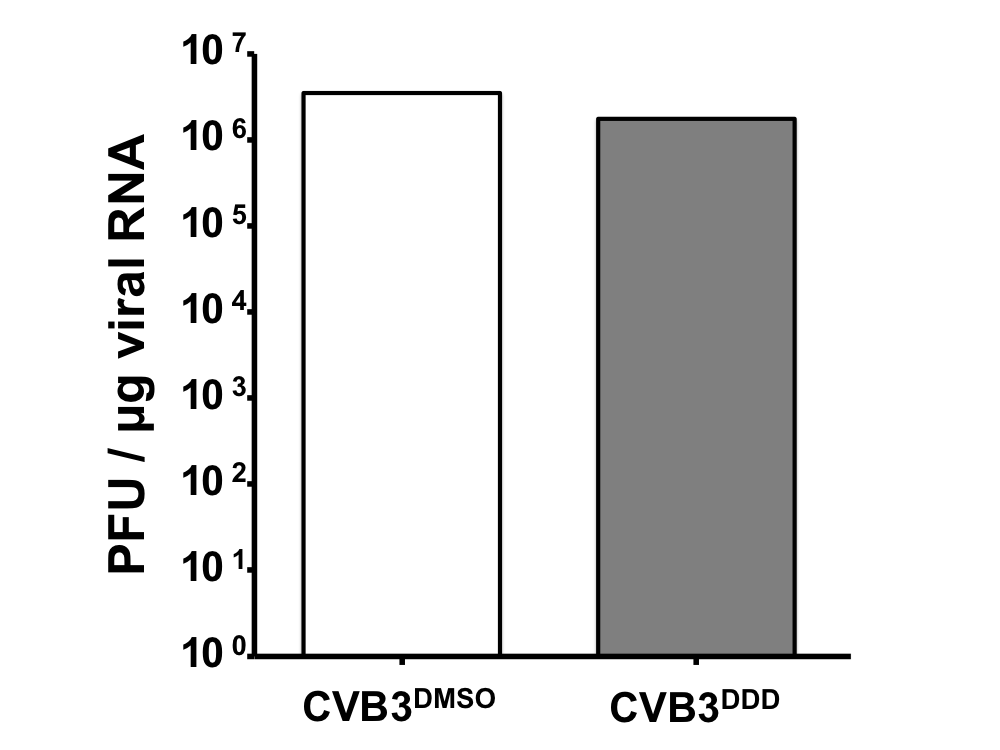

Supplement: S6 Fig — HeLa cells were transfected with equal amounts of viral genomic RNA extracted from purified CVB3DDD and CVB3DMSO particles obtained by propagation of CVB3 in HeLa Ohio in presence of 5 μM DDD85646 or DMSO (solvent control). Cell lysates prepared 60 h post transfection were used to determine virus yield by end point dilution as the 50% tissue culture infective dose (TCID50) per ml. Shown on the y-axis of the bar plot is the specific infectivity obtained for CVB3DMSO and CVB3DDD RNA, calculated from the data as the number of PFU (= TCID50 x 0.7) per μg transfected viral RNA genomes. (TIF) [file ppat.1007203.s007.tif]

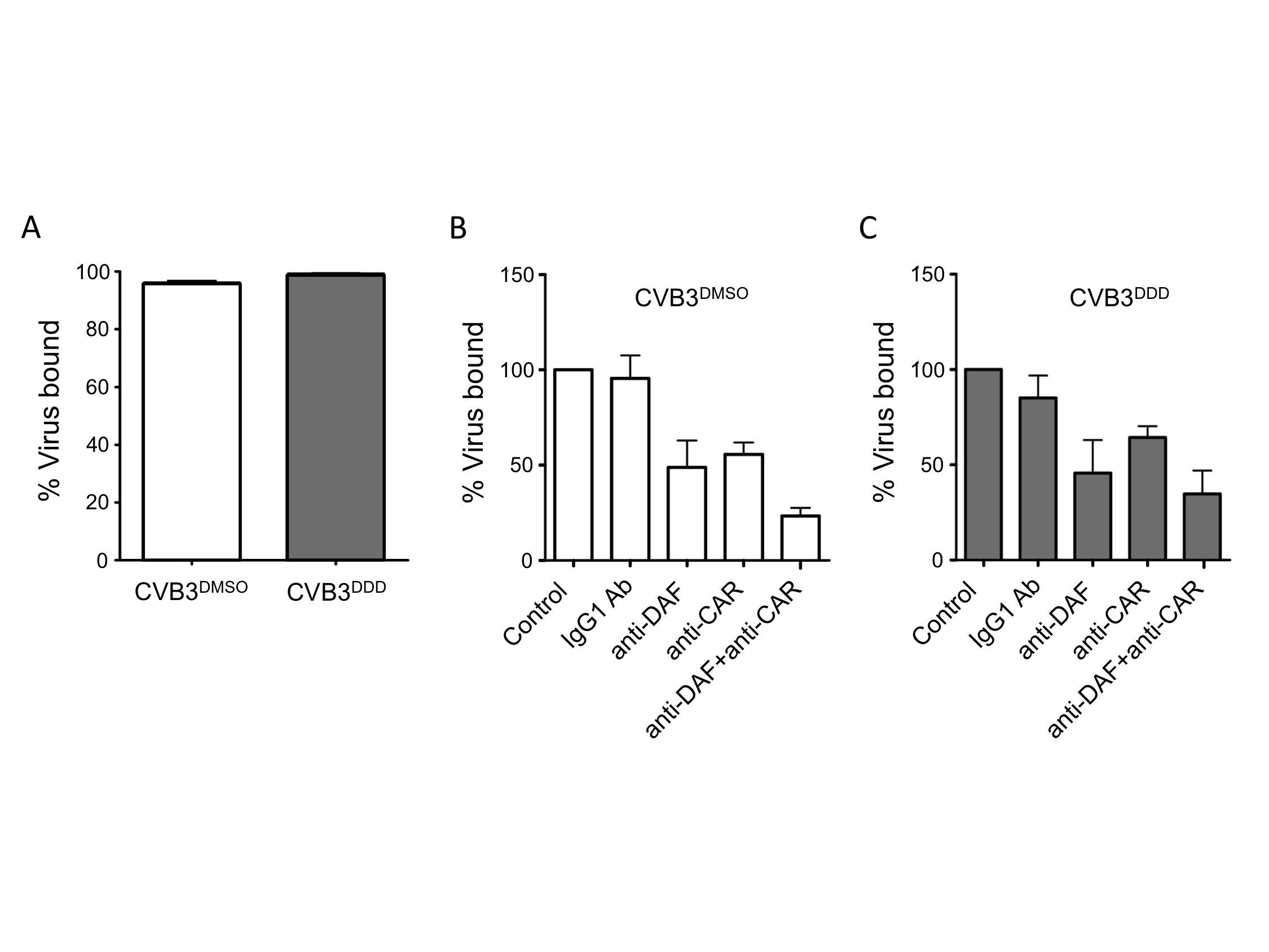

Supplement: S7 Fig — (A) Equal amounts of CVB3DDD and CVB3DMSO (obtained by propagation of CVB3 in HeLa cells in presence of 5 μM DDD85646 or DMSO as solvent control) quantified by RT-qPCR as SuperNuclease protected genomes (corresponding to an MOI of 1 for CVB3DMSO) were added to HeLa cells grown in 24-well plates and allowed to attach for 1 h at 4°C. Cells were washed with PBS and the amount of cell-associated viral RNA genomes was measured by RT-qPCR and normalized to the analogously determined quantity of GAPDH mRNA. Each bar represents the mean ± SD, n = 4. (B) HeLa cells were preincubated for 1 h at 4°C with anti-CAR monoclonal antibody (clone RmcB), anti-DAF monoclonal antibody (clone BRIC 216), anti-CAR + anti-DAF monoclonal antibodies, or mouse IgG1 isotype-control monoclonal antibody (each at 10 μg/ml), or left untreated. Cells were rinsed with PBS and equal amounts in genomes/cell of CVB3DMSO or (C) CVB3DDD (corresponding to an MOI of 1 for the former) were added and incubation continued for 1 h at 4°C. Cells were washed with PBS and cell-associated viral RNA genomes were measured and normalized to GAPDH mRNA as in (A). Data are displayed as percent binding of CVB3DDD or CVB3DMSO in presence of the antibodies relative to the untreated control set to 100%. Each bar represents the mean ± SD, n = 4. (TIF) [file ppat.1007203.s008.tif]

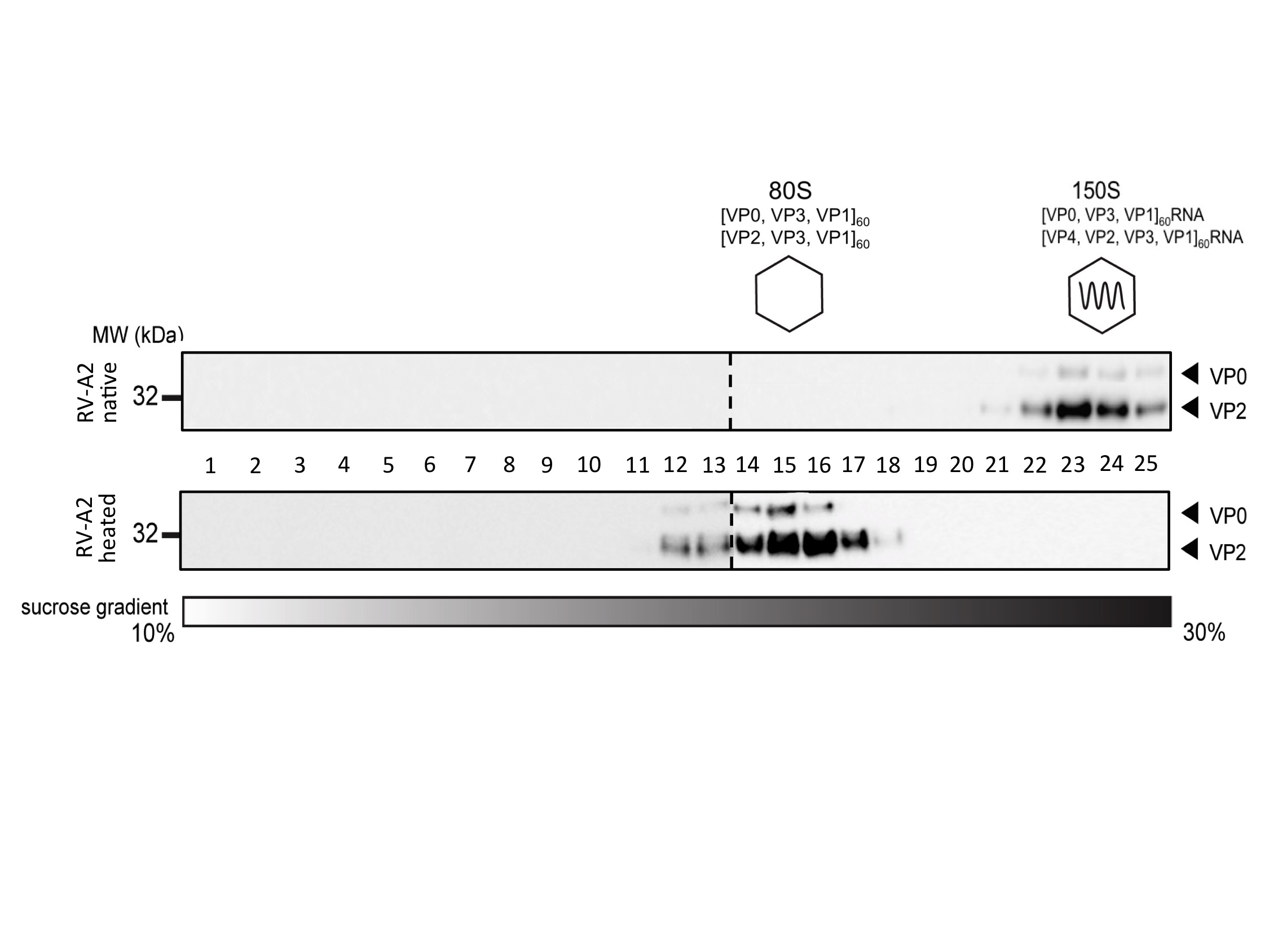

Supplement: S8 Fig — Highly pure samples of RV-A2 150S native (full) and 80S (empty) subviral particles (devoid of RNA and VP4, obtained by heating of native virus for 30 min at 55°C) were each sedimented through a 10–30% (w/v) sucrose density gradient. Fractions (200 μl) taken from top to bottom were analyzed for the presence of full 150S (top panel) and empty 80S particles (bottom panel) by Western blotting using the VP2/VP0-specific monoclonal antibody 8F5. The presence of a weak VP0 band in both blots results from the small amounts of 150S provirions in the native virus preparation, which also transform into 80S particles upon heating of the sample by expelling their genomic RNA (but not VP4 being part of VP0). The sucrose density expressed as g/cm3 is depicted at the bottom. In each instance images from two individual Western blots have been stitched together (indicated by the short-dashed line) for better appreciation of the results. The numbers between the two blots indicate the fractions (1, top to 25, bottom) examined for the sedimentation range of RV-A2 80S or 150S particles (displayed as cartoons and corresponding composition above their respective peak fractions). The corresponding upper and lower bound fractions were used to define the equivalent sedimentation range for the 75S and 150S particles of CVB3DDD and CVB3DMSO, centrifuged under identical conditions and similarly revealed by immunoblotting with anti-VP2/VP0 and VP1 specific antibodies (Fig 7B). (TIF) [file ppat.1007203.s009.tif]

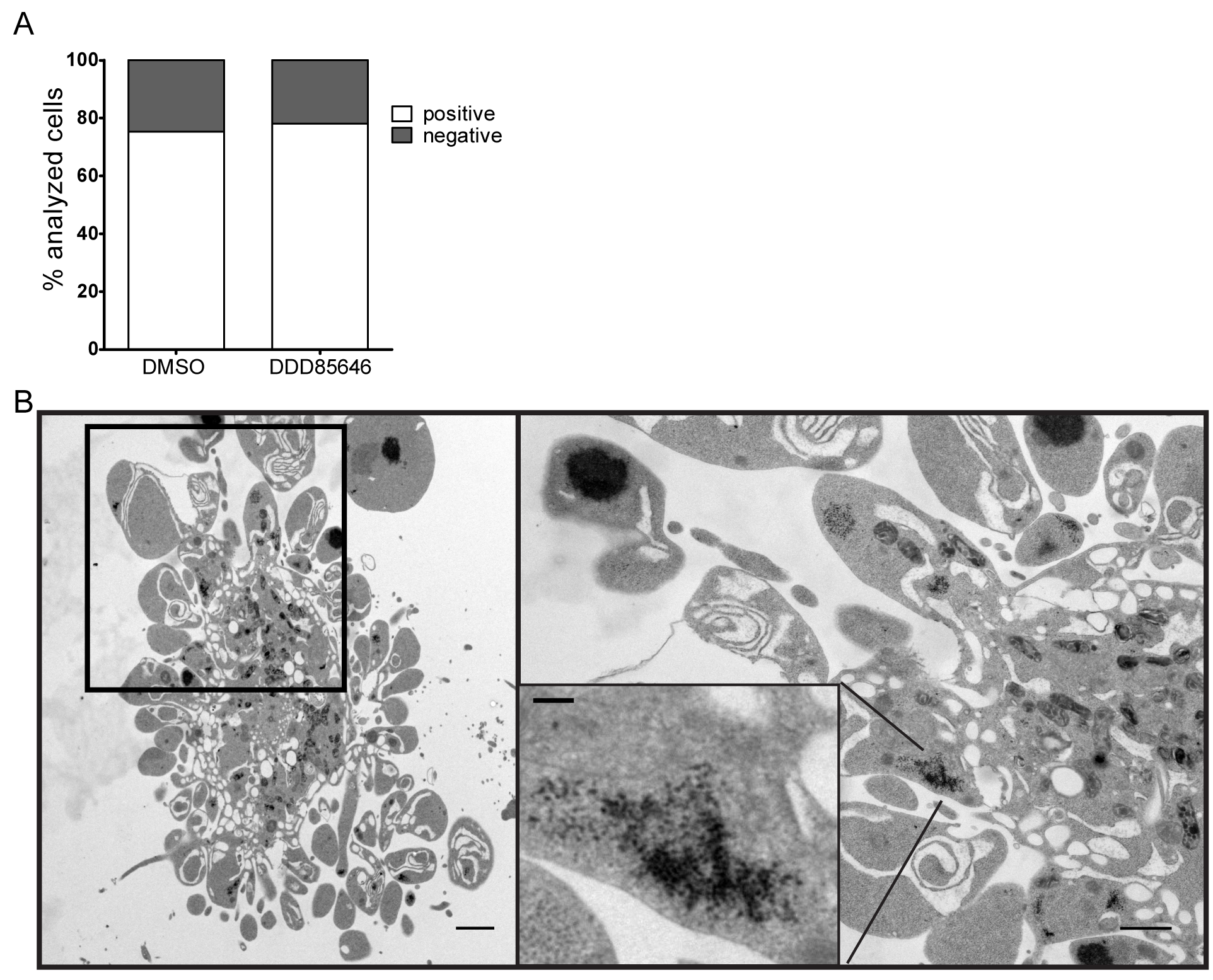

Supplement: S9 Fig — (A) Bar-graphs displaying the fraction of cells, which exhibit no or one to several virus blebs in thin sections of HeLa cells at 7 h p.i. upon challenge with CVB3 in presence of 5 μM DDD85646 or the DMSO solvent control. (B) TEM image showing a fragmented apoptotic CVB3-infected cell (MOI of 10) exhibiting multiple cytoplasmic protrusions filled with arrayed virus particles. (TIF) [file ppat.1007203.s010.tif]

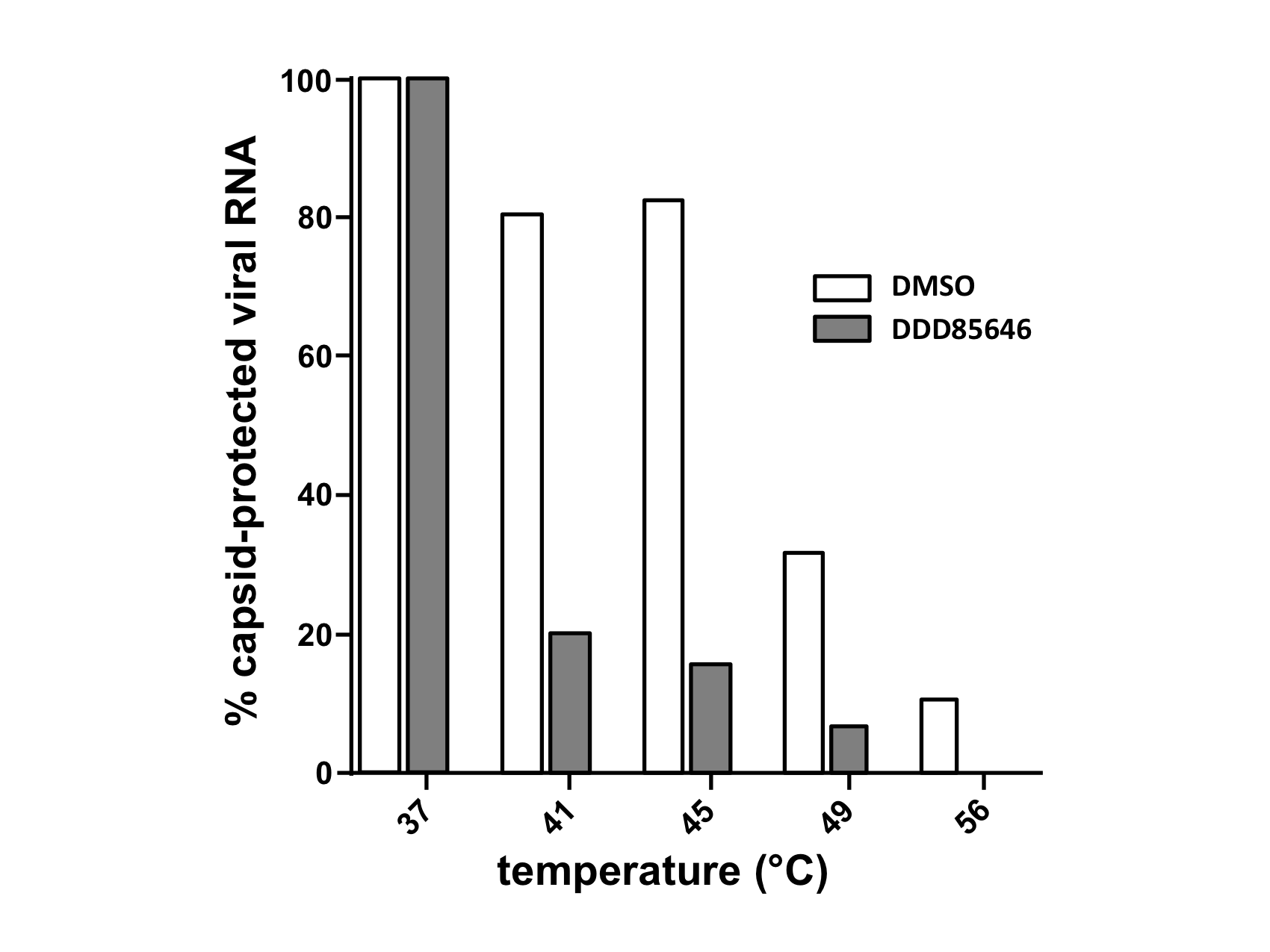

Supplement: S10 Fig — Aliquots (100 μl) of CVB3DDD or CVB3DMSO at 109 genomes/ml were incubated at 37, 41, 45, 49, and 56°C for 30 min to thermally trigger viral RNA uncoating. The samples were incubated with SuperNuclease to digest the released genomes; the remaining capsid-protected viral genomes were extracted with TRIzol and quantified by RT-qPCR. The percentage was plotted against the incubation temperature (°C) with 100% assigned to the value obtained for the 37°C control sample. CVB3DMSO particles most extensively uncoat between 45–49°C, while about 80% of the CVB3DDD particles (likely the fragile provirions) release their RNA genome already between 37–41°C; the remaining 20% (likely mostly the about fivefold under-represented, more stable myr-deficient virions) uncoat over a similar temperature window as CVB3DMSO. (TIF) [file ppat.1007203.s011.tif]

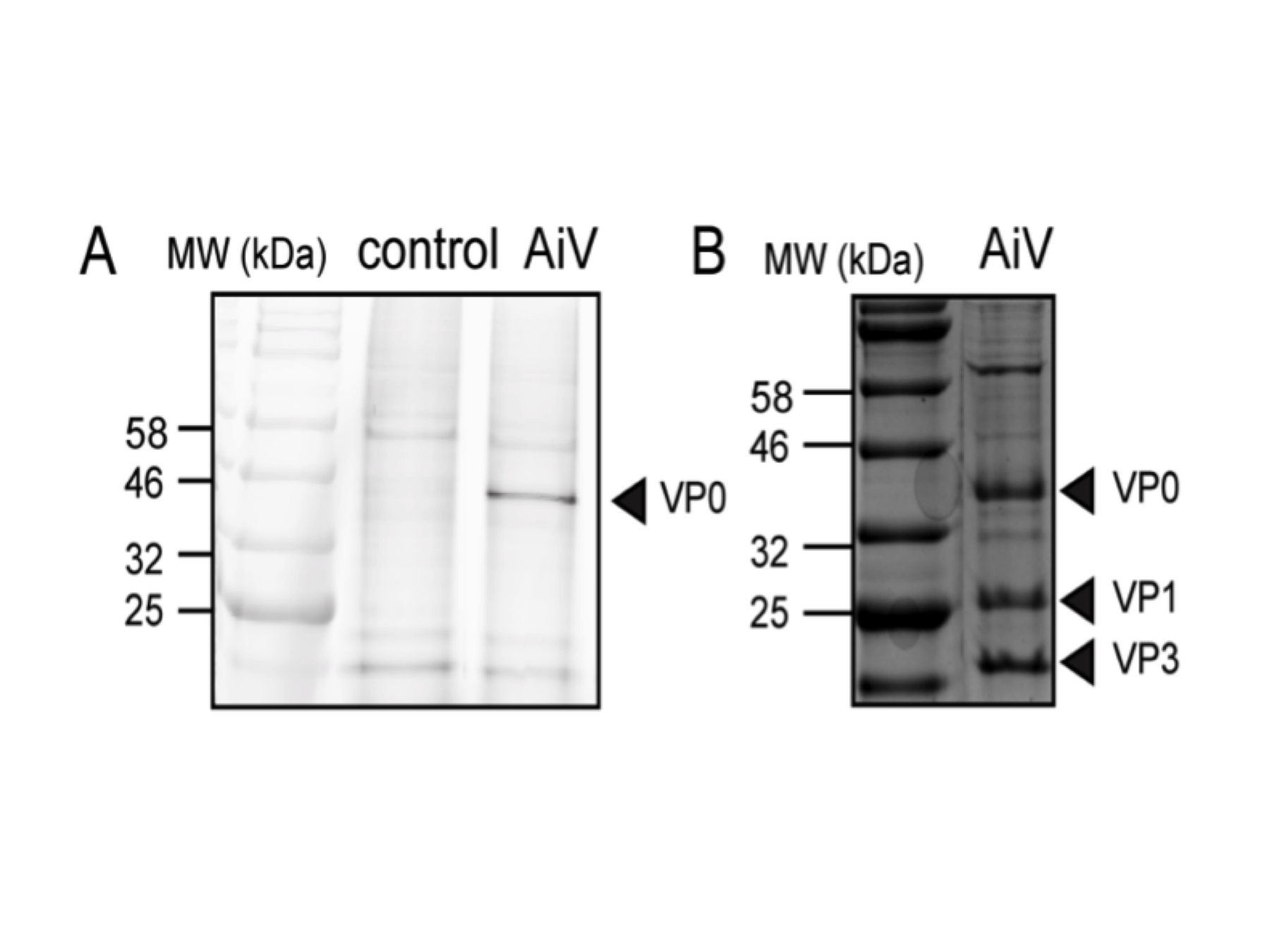

Supplement: S11 Fig — (A) Vero cells were either sham-infected or infected with AiV-1 at an MOI of 10. The myristic acid analogue Az-12 was added 4 h p.i. and incubation continued for 6 h to allow metabolic incorporation into viral proteins. Cells were lysed 10 h p.i., Az-12-bearing proteins were ligated to the fluorescent reporter Cy5.5-alkyne by click chemistry, separated by 15% SDS-PAGE and visualized via in-gel fluorescence. This revealed a band at ~ 40 kDa uniquely present in the extract from virus-infected cells (right lane compared to the non-infected control (left lane)), close to the Mr of 38.9 kDa predicted for VP0 of AiV-1. (B) A Coomassie blue-stained band at the same molecular weight corresponding to VP0 is present in a highly purified Aichi virus 1 sample run in parallel. (TIF) [file ppat.1007203.s012.tif]
