# Supplementary material for: Cellular N-myristoyltransferases play a crucial picornavirus genus-specific role in viral assembly, virion maturation, and infectivity
Source: PLoS Pathog. 2018 Aug 6;14(8):e1007203. doi: 10.1371/journal.ppat.1007203 (PMC6089459; doi:10.1371/journal.ppat.1007203)
Supplement: S12 Fig — (PDF) [file ppat.1007203.s013.pdf]

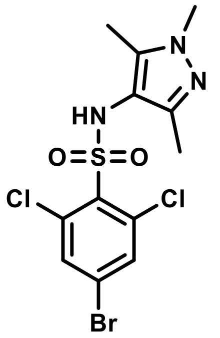

4-Bromo-2,6-dichloro-*N*-(1,3,5-trimethyl-1*H*-pyrazol-4-yl)benzenesulfonamide was synthesized according to literature [1]. ^{1}H NMR (400 MHz, DMSO-d_6) δ 9.70 (s, 1H), 7.96 (s, 2H), 3.56 (s, 3H), 1.91 (s, 3H), 1.71 (s, 3H). ^{13}C NMR (101 MHz, DMSO) δ 143.8, 137.2, 135.4, 135.2, 133.8, 125.9, 111.6, 36.2, 10.4, 8.5.

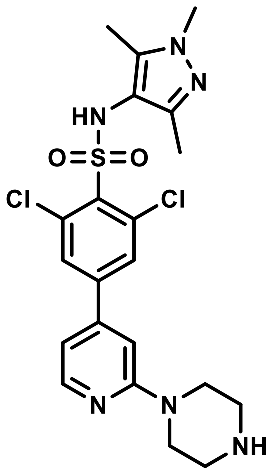

2,6-Dichloro-4-(2-(piperazin-1-yl)pyridin-4-yl)-*N*-(1,3,5-trimethyl-1*H*-pyrazol-4-yl)benzenesulfonamide (DDD85646) was synthesized according to literature [2]. ^{1}H NMR (400 MHz, $CDCl_3$) δ 8.27 (d, *J* = 5.2 Hz, 1H), 7.64 (s, 2H), 6.75 (d, *J* = 5.1 Hz, 1H), 6.70 (s, 1H), 3.66 (s, 3H), 3.63 – 3.57 (m, 4H), 3.04 – 2.99 (m, 4H), 2.16 (s, 3H), 1.76 (s, 3H). ^{13}C NMR (101 MHz, $CDCl_3$) δ 160.2, 149.1, 145.3, 144.8, 144.2, 138.8, 135.9, 135.6, 129.5, 111.8, 111.0, 104.5, 46.1, 45.7, 36.6, 10.6, 9.4.

1. Frearson JA, Brand S, McElroy SP, Cleghorn LA, Smid O, Stojanovski L, et al. N-myristoyltransferase inhibitors as new leads to treat sleeping sickness. Nature. 2010;464(7289):728-32. Epub 2010/04/03. doi: 10.1038/nature08893. PubMed PMID: 20360736; PubMed Central PMCID: PMC2917743.

2. Brand S, Cleghorn LA, McElroy SP, Robinson DA, Smith VC, Hallyburton I, et al. Discovery of a novel class of orally active trypanocidal N-myristoyltransferase inhibitors. J Med Chem. 2012;55(1):140-52. doi: 10.1021/jm201091t. PubMed PMID: 22148754; PubMed Central PMCID: PMCPMC3256935.

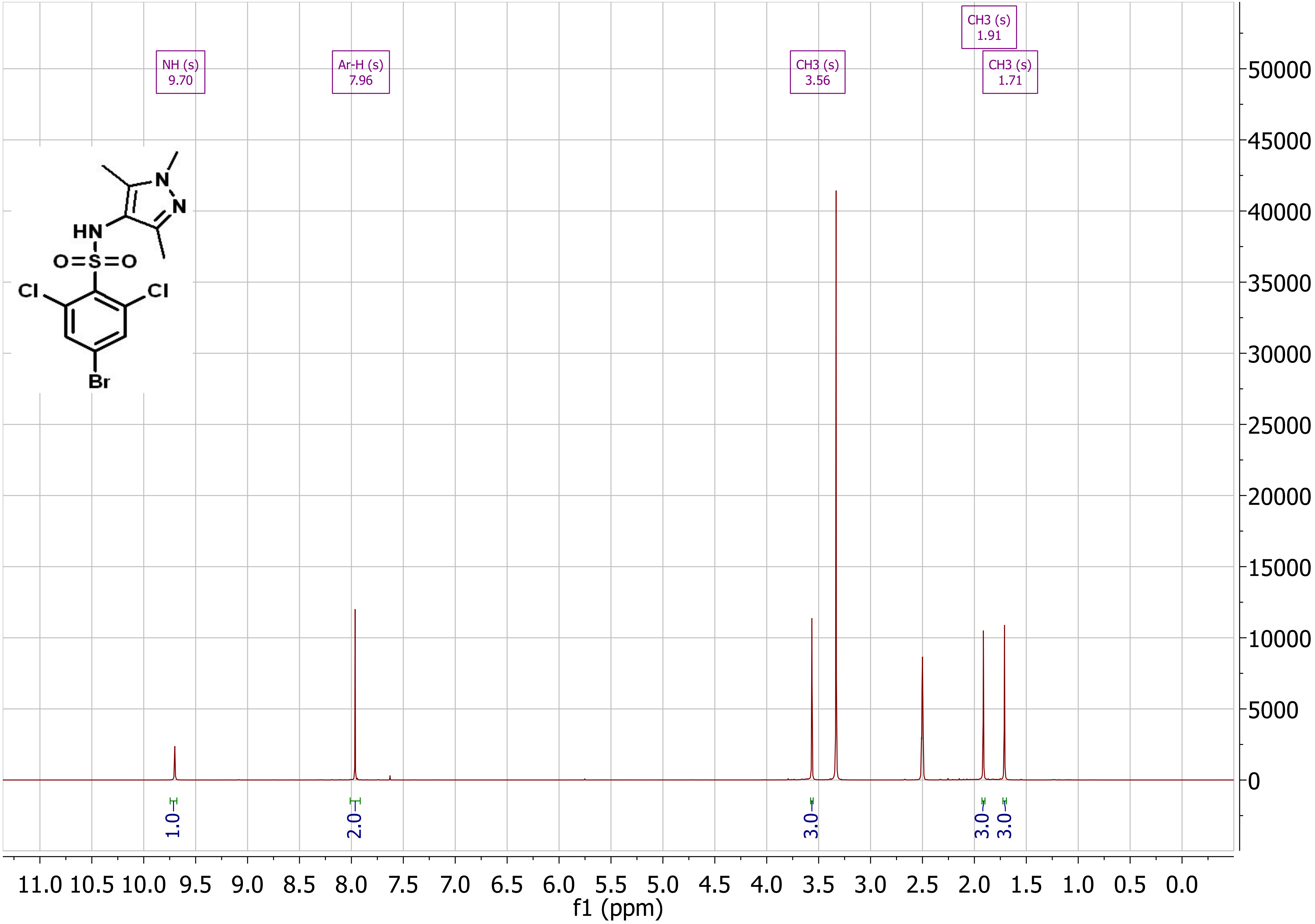

NH (s)
9.70
Ar-H (s)
7.96
CH3 (s)
3.56
CH3 (s)
1.91
CH3 (s)
1.71
N
N
HN
O=S=O
Cl
Cl
Br
1.0
2.0
3.0
3.0
3.0
50000
45000
40000
35000
30000
25000
20000
15000
10000
5000
0
11.0 10.5 10.0 9.5 9.0 8.5 8.0 7.5 7.0 6.5 6.0 5.5 5.0 4.5 4.0 3.5 3.0 2.5 2.0 1.5 1.0 0.5 0.0
f1 (ppm)

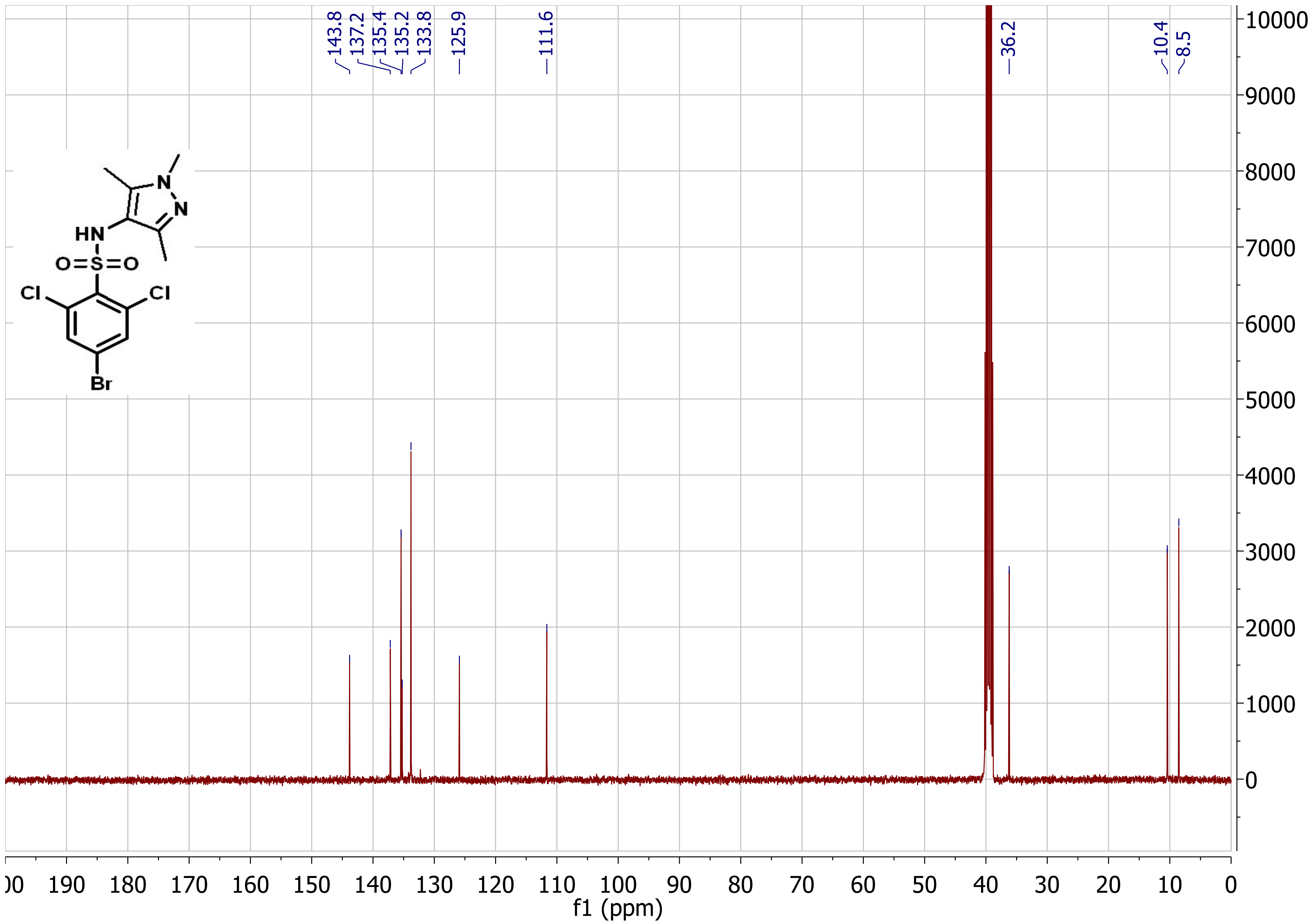
143.8
137.2
135.4
135.2
133.8
125.9
111.6
36.2
10.4
8.5
HN
N
N
O=S=O
Cl
Cl
Br
10000
9000
8000
7000
6000
5000
4000
3000
2000
1000
0
190
180
170
160
150
140
130
120
110
100
90
80
70
60
50
40
30
20
10
0
f1 (ppm)

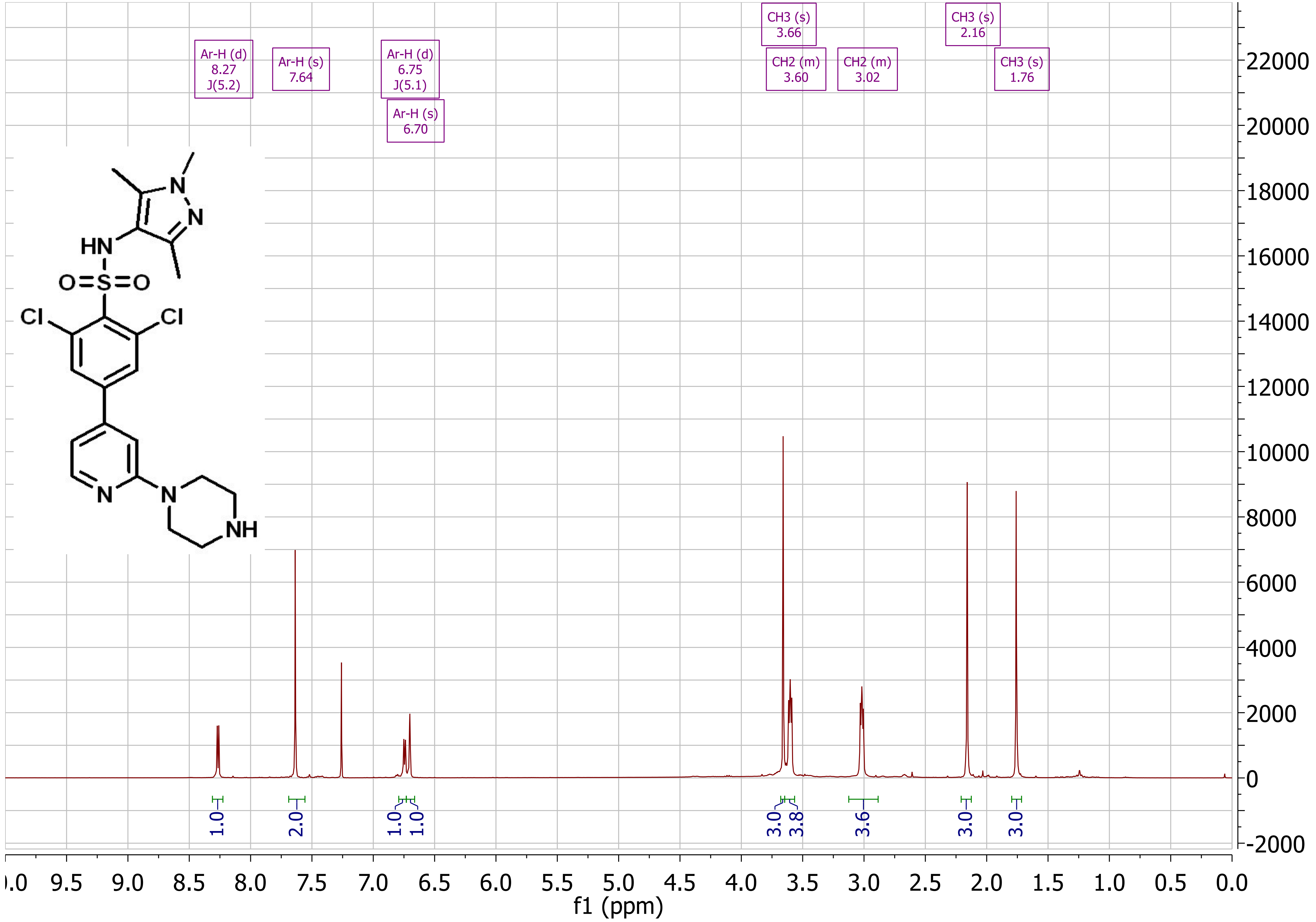

Ar-H (d)
8.27
J(5.2)
Ar-H (s)
7.64
Ar-H (d)
6.75
J(5.1)
Ar-H (s)
6.70
CH3 (s)
3.66
CH2 (m)
3.60
CH2 (m)
3.02
CH3 (s)
2.16
CH3 (s)
1.76
HN
O=S=O
Cl
Cl
N
N
N
N
NH
1.0
2.0
1.0
1.0
3.0
3.8
3.6
3.0
3.0
22000
20000
18000
16000
14000
12000
10000
8000
6000
4000
2000
0
-2000
9.5
9.0
8.5
8.0
7.5
7.0
6.5
6.0
5.5
5.0
4.5
4.0
3.5
3.0
2.5
2.0
1.5
1.0
0.5
0.0
f1 (ppm)

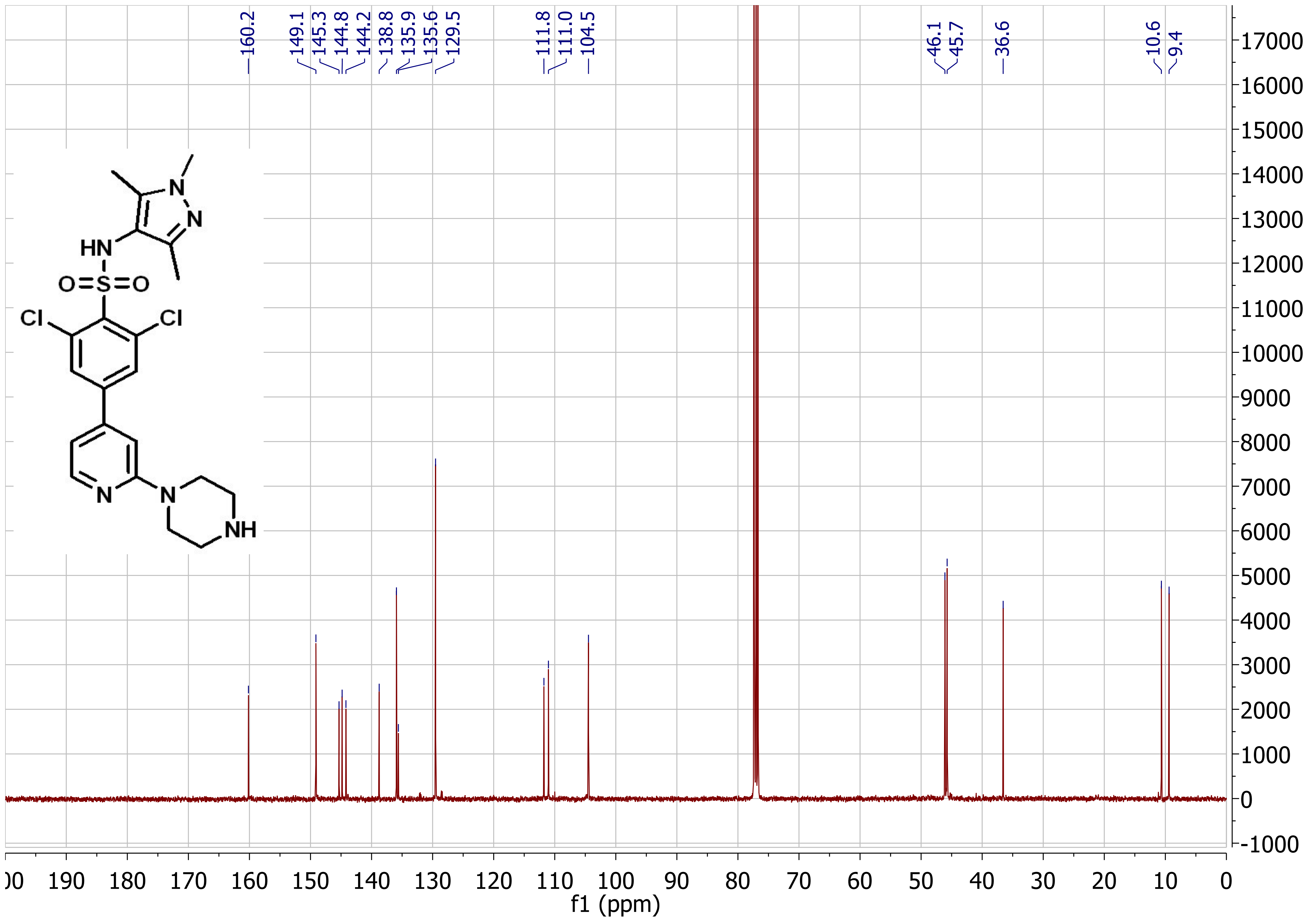

160.2
149.1
145.3
144.8
144.2
138.8
135.9
135.6
129.5
111.8
111.0
104.5
46.1
45.7
36.6
10.6
9.4
HN
O=S=O
Cl
Cl
N
N
N
N
NH
f1 (ppm)
